# Recent Advances in Designing and Fabricating Self‐Supported Nanoelectrodes for Supercapacitors

**DOI:** 10.1002/advs.201700188

**Published:** 2017-07-10

**Authors:** Huaping Zhao, Long Liu, Ranjith Vellacheri, Yong Lei

**Affiliations:** ^1^ Institute of Physics & IMN Macronano^®^ Ilmenau University of Technology Ilmenau 98693 Germany

**Keywords:** heterogeneous nanoelectrodes, homogeneous nanoelectrodes, self‐supported nanoarrays, supercapacitors

## Abstract

Owing to the outstanding advantages as electrical energy storage system, supercapacitors have attracted tremendous research interests over the past decade. Current research efforts are being devoted to improve the energy storage capabilities of supercapacitors through either discovering novel electroactive materials or nanostructuring existing electroactive materials. From the device point of view, the energy storage performance of supercapacitor not only depends on the electroactive materials themselves, but importantly, relies on the structure of electrode whether it allows the electroactive materials to reach their full potentials for energy storage. With respect to utilizing nanostructured electroactive materials, the key issue is to retain all advantages of the nanoscale features for supercapacitors when being assembled into electrodes and the following devices. Rational design and fabrication of self‐supported nanoelectrodes is therefore considered as the most promising strategy to address this challenge. In this review, we summarize the recent advances in designing and fabricating self‐supported nanoelectrodes for supercapacitors towards high energy storage capability. Self‐supported homogeneous and heterogeneous nanoelectrodes in the forms of one‐dimensional (1D) nanoarrays, two‐dimensional (2D) nanoarrays, and three‐dimensional (3D) nanoporous architectures are introduced with their representative results presented. The challenges and perspectives in this field are also discussed.

## 1 Introduction

With the over‐reliance on fossil fuels and the gradual deterioration of global climate and environment situations due to the emissions of greenhouse gases from these combustion sources, extensive research efforts have currently been dedicated to develop clean and sustainable energy resources such as solar and wind power to combat climate change and to meet the ever‐increasing energy demand. Actually, in recent years, there is already quite a few impressive progress in developing wind turbines as well as solar energy harvesting technologies for converting solar energy to electricity either via solar thermal or photovoltaic approaches. However, both wind and solar energy resources inevitably have intermittent and instability characteristics, and thus their intensive utilization highlights the increasing importance of the matched energy storage technologies. In this regard, electrochemical energy storage devices such as batteries and supercapacitors are strongly recommended to be one of the most promising energy storage technologies for effectively harvesting these intermittent energy sources.^1^, ^2^, ^3^, ^4^, ^5^

Particularly, supercapacitors (so‐called electrochemical capacitors) are of great current concern, attributing to their much higher power densities (>500 W kg^–1^), more excellent reversibility, and much longer cycle life (>10^5^ cycles) than rechargeable batteries. Different from batteries, supercapacitors store energy by means of either adsorption‐desorption of electrolyte ions on the electrode surface (electric double layer capacitor, EDLC), or fast and reversible surface redox reactions between electroactive materials and electrolyte ions (pseudocapacitors), which charge storage are happened at the electrode/electrolyte interface in the both cases. Therefore, in contrast to batteries, charging of supercapacitor is not limited by diffusion of ions in the bulk of the electrode materials, and hence higher power densities can be achieved in supercapacitors.^6^ However, the major challenge for supercapacitors, when compared to batteries, is their insufficient energy density, which hinders their application scopes especially when high energy density is required. To address this challenge, tremendous research efforts have been devoted to increasing the energy density of supercapacitors, without sacrificing high power capability, to be close to or even beyond batteries.^7^, ^8^, ^9^ As known, the energy density of supercapacitor is in proportion to both the capacitance (*C*) and the cell potential (*V*), that is *E* = *0.5CV^2^*. Therefore, the energy density can be efficiently improved by increasing either or both of the capacitance and the cell potential. Aiming at increasing capacitance, the electroactive materials with high specific surface area and the pseudocapacitive materials (e.g., some transition metal oxides/hydroxides and conducting polymers) with high specific capacitance are both advantageous to reach this goal, while enlarging the cell potential is typically achieved through either the development of novel electrolytes (e.g., organic electrolytes, gel polymer electrolytes, and ionic liquid electrolytes) or the adoption of asymmetric electrode configuration in supercapacitors.

As mentioned above, the strategies to increase the capacitance are either or both to enlarge the surface area of electroactive materials and/or to employ pseudocapacitive materials. Due to the non‐Faradaic processes (capacitive) in EDLC, the capacitance of EDLC largely depends on the surface area of electroactive materials, mainly carbonaceous materials such as activated carbon. Not only for EDLC materials, the high specific surface area of the pseudocapacitive materials allows more materials availability for the interfacial Faradaic electrochemical reactions, and thus more charges will be stored, resulting in high energy density. Nanomaterials are known to take on peculiar properties compared to the bulk materials, in particular, nanomaterials have an extraordinarily high surface area to volume ratio, making more electroactive materials accessible to the electrolyte for double‐layer formation and/or revisable Faradaic reactions. Therefore, the utilization of nanomaterials are more preferred to increase the energy density of supercapacitors.^10^, ^11^, ^12^ In the past decades, major research effort has been accordingly performed to synthesize different nanostructured electroactive materials with different methods for constructing supercapacitor electrodes and devices with considerable achievements.^13^, ^14^, ^15^, ^16^ As we all know, a full supercapacitor device generally has four key components: positive and negative electrodes, separator, and electrolyte, and thus the overall performance of supercapacitor depends on all the above four key components. Regarding the electrodes, it consists of electroactive materials and current collector, in which electroactive materials are responsible for charge storage and current collector takes charge of electron transfer. Apart from electroactive materials responsible for energy storage, the architecture of electrode also plays a significant role in determining the electroactive materials to reach their full potentials in charge storage, and this issue is particularly prominent in supercapacitor nanoelectrodes.

**Scheme 1:**
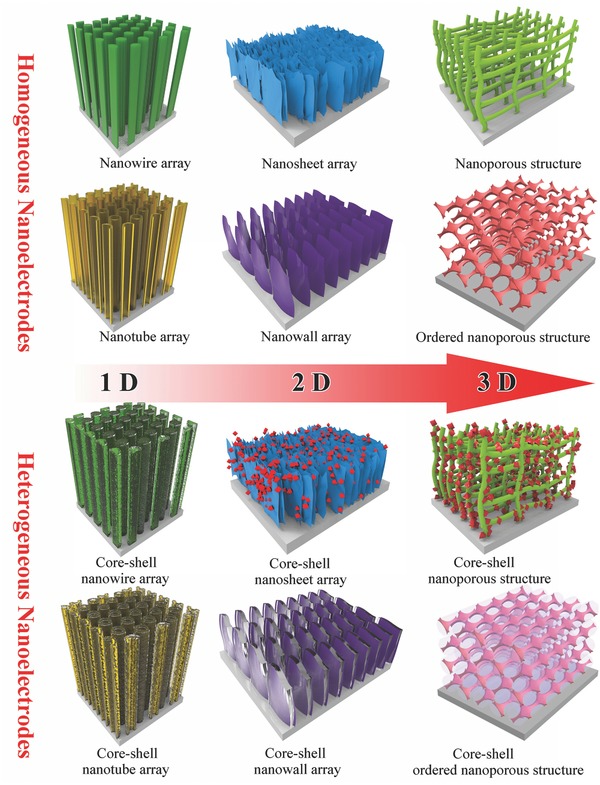
Schematically illustrating the typical geometries of self‐supported homogeneous and heterogeneous nanoelectrodes for supercapacitors.

Remarkably, the merits of nanomaterials would bring fully beneficial effects if their merits could be retained when the nanomaterials are assembled into a structure capable of being used as a nanoelectrode. To this end, one of the important principles for designing supercapacitor nanoelectrodes is to ensure efficient surface area of the nanostructured electroactive materials as large as possible, which electrolyte could easily and rapidly access. Larger accessible surface area the electroactive materials will have, more charges the electrodes will be able to store. So far, nanostructured electroactive materials are commonly synthesized into the form of either powder or free‐standing arrays. The most outstanding feature of nanostructured electroactive materials in powdery form is the synthesis easiness in large scale, however, binders and conductive additives are certainly necessary in use when fabricating supercapacitor electrodes mostly with slurry‐casting method. Nonetheless, with the use of binders and conductive additives, the fabricated electrodes would not be able to make the best use of the large surface area of nanomaterials and meanwhile will simultaneously generate some “dead volume” in the fabricated electrodes which is not accessible for electrolyte permeating, resulting in low utilization ratio of electroactive materials.^17^ In contrast to powdery nanomaterials, self‐supported nanoarrays of electroactive materials growth on a conductive substrate are considered to be highly desirable electrode architecture without the necessary addition of binders and conductive additives, namely binder‐free electrodes. Such self‐supported nanoelectrodes provide unique benefits over the slurry‐casting electrodes such as improved utilization of electroactive materials and enhanced charge/ion transport efficiency. An intensive review has particularly summarized the advantages of the hybrid nanostructure arrays of metal oxides as the binder‐free electrodes for electrochemical energy storage.^18^ Accordingly, it can be generally considered that the real nanoelectrodes for supercapacitor should be mainly referred to various self‐supported nanoarrays of electroactive materials grown on conductive substrates. The most common method for producing supercapacitor nanoelectrodes is to directly fabricate electroactive materials into the forms of free‐standing either one‐dimensional (1D) nanoarrays (such as nanowires, nanorods, and nanotubes), or two‐dimensional (2D) nanoarrays (such as nanosheets, nanowalls, and nanoflakes), or three‐dimensional (3D) nanoporous architectures on conductive substrates (**Scheme** **1**) through different nanostructuring techniques, including electrochemical deposition, hydrothermal/solvothermal method, anodization, atomic layer deposition (ALD), chemical vapor deposition, chemical bath deposition, and electrospinning, etc. Here, the conductive substrates are usually metal foils, metal foams, metal wires, carbon fabrics, and other conductive substrates, and they functionalize as not only the supporters but also the current collectors. Considering that such kind of supercapacitor nanoelectrodes is nanoarrays of only single electroactive material in most cases, they can be classified as self‐supported homogeneous nanoelectrodes (Scheme 1). In addition to homogeneous nanoelectrodes, there are also self‐supported heterogeneous nanoelectrodes for supercapacitors that is nanoarrays consisting of either electroactive materials with nanostructured current collectors/supporters or more than one kinds of electroactive materials in single nanoelectrodes. In general, self‐supported heterogeneous nanoelectrodes adopt a core‐shell configuration in which a shell of electroactive materials is conformally deposited on a core of free‐standing nanostructured current collectors or another kind of electroactive materials that are grown on a conductive substrate (Scheme 1).^19^ And it is also worth pointing out that both homogeneous and heterogeneous nanoelectrodes are basically fabricated through either template‐assisted or template‐free methods. In recent years, both homogeneous and heterogeneous self‐supported nanoelectrodes have been rationally designed and produced for achieving more noteworthy improvements in supercapacitor performances.

In this review, we summarize the recent advances in designing and fabricating self‐supported nanoelectrodes for supercapacitors towards high energy storage capability. The typical morphologies and supercapacitor performance of self‐supported homogeneous and heterogeneous nanoelectrodes produced through the representative template‐free or template‐assisted methods are mainly discussed, but the comparison of different fabrication techniques are not included in this review. This review is organized as follows: we will first describe the representative progress in studying nanoarrays of electroactive materials as self‐supported homogeneous nanoelectrodes for supercapacitors, with the representative examples including 1D nanoarrays (e.g., nanowires, nanorods, and nanotubes) and 2D nanoarrays (e.g., nanosheets, nanowalls, and nanoflakes) as well as 3D nanoporous architectures illustrated. Then we will discuss the recent development of self‐supported nanostructured current collectors or supporters for constructing self‐supported heterogeneous supercapacitors nanoelectrodes, and discuss their advantages for supercapacitors. Finally, we will summarize these achievements of self‐supported nanoelectrodes for supercapacitors, and suggest the challenges and future perspectives in this field.

## 2 Self‐Supported Homogeneous Nanoelectrodes

As known, to be an ideal nanoelectrode for supercapacitors, it should have higher specific surface area for storing more charges and meanwhile lower ion/electron transport resistance for realizing high rate capability. In general, the direct way for fabricating self‐supported nanoelectrodes is to synthesize nanoarrays of electroactive materials on a conductive substrate. These obtained nanoarrays are homogeneous with mostly only electroactive material in the form of arrayed 1D, or 2D, or 3D nanostructures, and accordingly the homogeneous supercapacitor nanoelectrodes can be geometrically categorized into three kinds: 1D nanoarrays, 2D nanoarrays, and 3D nanoporous architectures.

### 2.1 Self‐Supported Homogeneous 1D Nanoarrays

1D nanoarrays, such as nanowires, nanorods, and nanotubes, have been extensively investigated as building blocks for supercapacitors mainly due to the fact that the longitudinal axis of 1D nanostructures could provide efficient transport pathway for both electrons and ions, leading to high charge/discharge rate.^10^, ^20^, ^21^ Arrayed nanowires and nanotubes of electroactive materials are therefore the most typical self‐supported nanoelectrodes for supercapacitors. So far, the reported methods for synthesizing arrayed nanowires, nanorods, and nanotubes are generally included template‐assisted and template‐free methods. In the following, we will introduce some representative homogeneous nanoelectrodes of several typical electroactive materials fabricated through either template‐assisted or template‐free methods for supercapacitor applications.

#### 2.1.1 Template‐Assisted Method

**Figure 1:**
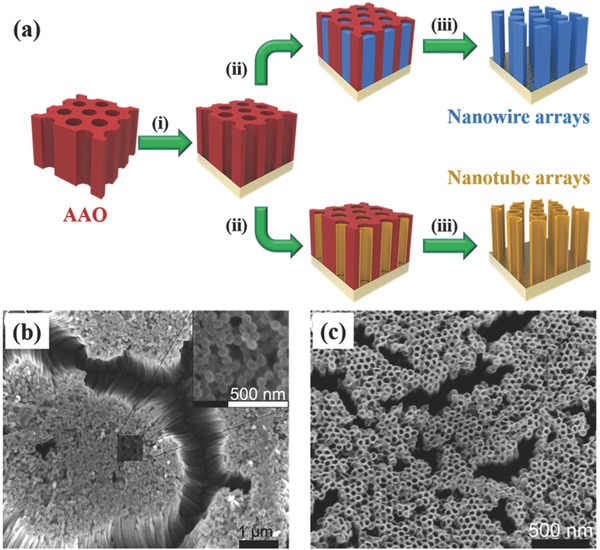
a) Schematically illustrating the typical process of AAO template‐assisted fabrication of 1D nanoarrays: (i) depositing conduction substrate; (ii) depositing electroactive materials; (iii) removing AAO template. SEM images of MnO_2_ (b) nanowire and (c) nanotube arrays fabricated through AAO template‐assisted electrochemical deposition. Reproduced with permission.^31^ Copyright 2014, AIP Publishing.

Owing to the strong versatility of classic porous anodic aluminum oxide (AAO) template‐assisted method, many kinds of electroactive materials have been fabricated into 1D nanoarrays with the forms of nanowires, nanorods, and nanotubes for supercapacitors through different techniques, including electrochemical deposition, atomic layer deposition (ALD) and chemical vapor deposition (CVD) as illustrated in **Figure** **1**a. Taking MnO_2_ as an example, Xu et al. prepared mesoporous nanowire arrays of MnO_2_ through AAO template‐assisted electrochemical deposition.^22^ The obtained mesoporous MnO_2_ nanowire arrays exhibited enhanced specific capacitance and charge/discharge performance, however, due to the intrinsically low conductivity of MnO_2_, the mesoporous MnO_2_ nanowire arrays still had much lower capacitance at a higher charge/discharge rate. Accordingly, Liu et al. fabricated MnO_2_/poly(3,4‐ethylenedioxythiophene) (PEDOT) coaxial nanowires by coelectrodeposition of MnO_2_ and PEDOT in a AAO template.^23^, ^24^ The core MnO_2_ provides high energy storage capacity, while the highly conductive and porous PEDOT shell facilitates the electron transport and ion diffusion into the core, thus the MnO_2_/PEDOT coaxial nanowires were found to have much better electrochemical energy storage performance than pure MnO_2_ nanowires. Importantly, the MnO_2_/PEDOT coaxial nanowire arrays could keep very high specific capacitances at high current densities. Furthermore, with MnO_2_/PEDOT coaxial nanowire arrays as cathode, the same group had assembled an asymmetric supercapacitor device together with PEDOT nanowire arrays as anode.^25^ Herein, the PEDOT nanowire arrays were also prepared by AAO template‐assisted electrochemical deposition. The conductive polymer PEDOT is also one kind of pseudocapacitive material, moreover, one important advantage of PEDOT as an anode material is its very large potential window. Therefore, the potential window of the asymmetrical device was allowed to be expanded to 1.7 V, benefiting to enhancing the energy density of the device. To further increase the energy storage capability of PEDOT nanowires as supercapacitor electrodes, they fabricated MnO_2_ nanoparticles loaded PEDOT nanowire arrays by soaking the AAO template‐directed PEDOT nanowires in potassium permanganate (KMnO_4_) solution. Compared to bare PEDOT nanowires, the inclusion of MnO_2_ nanoparticles had boost the energy storage capacity by 4 times, and the capacitance was even higher than that of MnO_2_/PEDOT coaxial nanowire arrays. Moreover, the MnO_2_ nanoparticles loaded PEDOT nanowire arrays exhibited well maintained capacitance even at high charge/discharge rate attributing to small dimensions of the PEDOT nanowires and MnO_2_ nanoparticles as well as the high conductivity of PEDOT. Beyond MnO_2_ and PEDOT, arrayed nanowires of some other electroactive materials had been produced and shown remarkable supercapacitor performance.^26^, ^27^, ^28^

In addition to having all the advantages of nanowires and nanorods, the nanotubular structure is more attractive as building block for constructing supercapacitor nanoelectrodes because of its much higher specific surface area than those of nanowire/nanorod, leading to a higher coefficient of utilization of electroactive materials, as well as the orderly pores in nanotube arrays to allow the fast ion diffusion process.^29^ Still taking MnO_2_ as an example, MnO_2_ nanotube arrays had been synthesized via electrochemical deposition with employing AAO as template (Figure 1c).^30^, ^31^ Attributing to the nanotubular architecture, the MnO_2_ nanotube array electrode exhibited outstanding capacitive behavior as compared to MnO_2_ nanowire array electrode. The specific capacitances for the MnO_2_ nanotube arrays were much higher than that of nanowire arrays, which was 320 F g^–1^ for nanotube arrays and only 101 F g^–1^ for nanowire arrays. With the same consideration and by employing the similar strategy, some other electroactive materials had also been synthesized into nanotubular structure such as RuO_2_,^32^ PEDOT,^33^ TiN,^34^ Co_3_O_4_−MnO_2_−NiO ternary compound,^35^ and carbon,^36^ etc. In particular, still by employing AAO template, Xue et al. recently designed novel wire‐shape graphene‐nanotube 3D architectures with a seamless nodal junction as excellent electrode architecture for all‐solid‐state cable‐type supercapacitors.^37^ They firstly prepared a wire with an aluminum core and an AAO shell by anodizing an aluminum wire in 0.3 m oxalic acid solution to convert the outside surface of an aluminum wire into an AAO shell. And further with the porous AAO shell as template, a graphitic carbon layer over the template surface, including the inner holes of the porous AAO shell, was deposited through a one‐step CVD process to finally produce the 3D open‐ended, radially aligned CNTs (RACNTs) sheathed with graphene. Here, the graphene layer can also be considered as a current collector for all CNTs in the structure of 3D RACNTs. Evidently, these as‐prepared 3D graphene‐RACNT fibers with a controllable surface area, meso/micropores, and superior electrical properties were found to be attractive electrodes for cable‐type supercapacitors. Especially, the open ends of the RACNTs on the aluminum wire surface could not only provide a large surface area but also allow for efficient diffusion of electrolytes to and from the constituent nanotubes. Subsequently, with the as‐prepared 3D graphene‐RACNT fibers as electrodes and poly(vinyl alcohol)/H_2_SO_4_ as the solid‐state electrolyte, all‐solid‐state cable‐type symmetric supercapacitors were fabricated and exhibited excellent supercapacitor performance with an areal specific capacitance of 3.33 mF cm^–2^ at the discharge current of 2.12 mA cm^–2^. Furthermore, a record‐high areal specific capacitance up to 89.4 mF cm^–2^ was obtained for the 3D graphene‐RACNT fiber (810 µm in diameter, in which the RACNTs shell thickness is ≈198 µm), which are among the best of the corresponding record‐high capacities reported for other cable‐type supercapacitors.^37^

**Figure 2:**
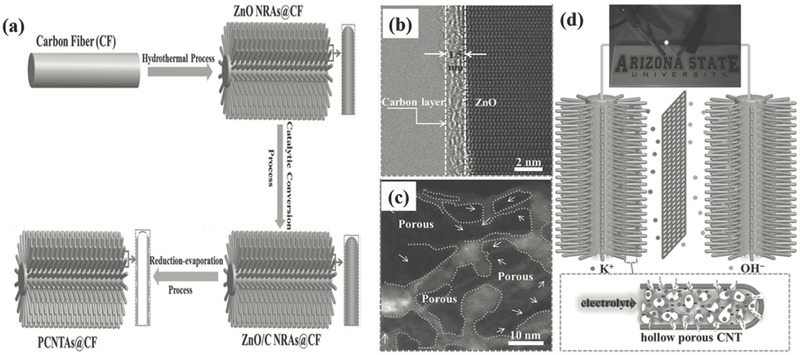
a) Schematic illustration of the fabrication procedures of PCNTAs@CF with ZnO nanorods as template. TEM images (b) and (c) revealing the porous structure of PCNTAs. d) Schematic illustration of multi‐transport pathways for both electrons and ions in PCNTAs. Reproduced with permission.^41^

Besides AAO as template, ZnO nanowires/nanorods are also widely used as nanostructuring templates to synthesize 1D nanoarrays of electroactive materials because of its good electronic conductivity and excellent chemical stability. More desirably, the ZnO core can be chemically removed by a weak acid or basic solution resulting in tubular nanostructures that retain the ZnO nanowire/nanorod morphology. By using ZnO nanorod arrays as sacrificial templates, open‐ended nanotube arrays of MnO_2_,^38^ Ni(OH)_2_,^39^ and polyaniline^40^ had been prepared for supercapacitor applications. Taking polyaniline as an example, ZnO nanorod arrays were firstly grown on Ti plate via hydrothermal reaction and then utilized as template for the following electrochemical polymerization of polyaniline. Finally, open‐ended polyaniline nanotube arrays were produced by dissolving ZnO nanorods in an ammonia solution. With this method, the inner diameter of polyaniline nanotubes depends on the diameter of ZnO nanorods, while the wall thickness of polyaniline nanotubes can be well controlled by adjusting the electropolymerization time. When employed as nanoelectrodes, the open‐ended hollow nanotubes allow electrolyte ions to readily penetrate into the interior of electrodes and access their internal surfaces, leading to an increase in efficient surface area for electrochemical reaction. Also utilizing ZnO nanorod arrays as template, Zhang et al. reported the synthesis of radially aligned porous carbon nanotube arrays (PCNTAs) on flexible carbon fibers.^41^ In their experiment, the PCNTAs were obtained by catalytic conversion of ethanol adsorbed on ZnO nanorod arrays hydrothermally grown on carbon fibers and then reduction‐evaporation of ZnO nanorods (**Figure** **2**a). The 3D arrangement, the diameters, and the lengths of the PCNTAs were precisely controlled by adjusting the synthesis protocols of the ZnO nanorod arrays. TEM characterization reveals the porous nature of the as‐obtained PCNTAs attributing to the removal of ZnO nanorods through reduction‐evaporation (Figure 2b and 2c). As a supercapacitor electrode, the spatially separated PCNTAs provide multiple channels for the diffusion and migration of the electrolyte ion during rapid charge/discharge processes, and importantly, the presence of mesopores on the walls and the large hollow interiors of the PCNTAs could provide not only fast transport of electrolyte ions but also high ion‐accessible surface areas for EDLC (Figure 2d). As a result, the symmetric supercapacitor device based on PCNTAs electrodes showed superior rate capability even with increasing scan rate up to 10 V s^–1^, indicating rapid current response to the change in voltage sweep as a result of fast ion diffusion in the electrodes and low interior electric resistance for the PCNTAs electrodes.

#### 2.1.2 Template‐Free Method

**Figure 3:**
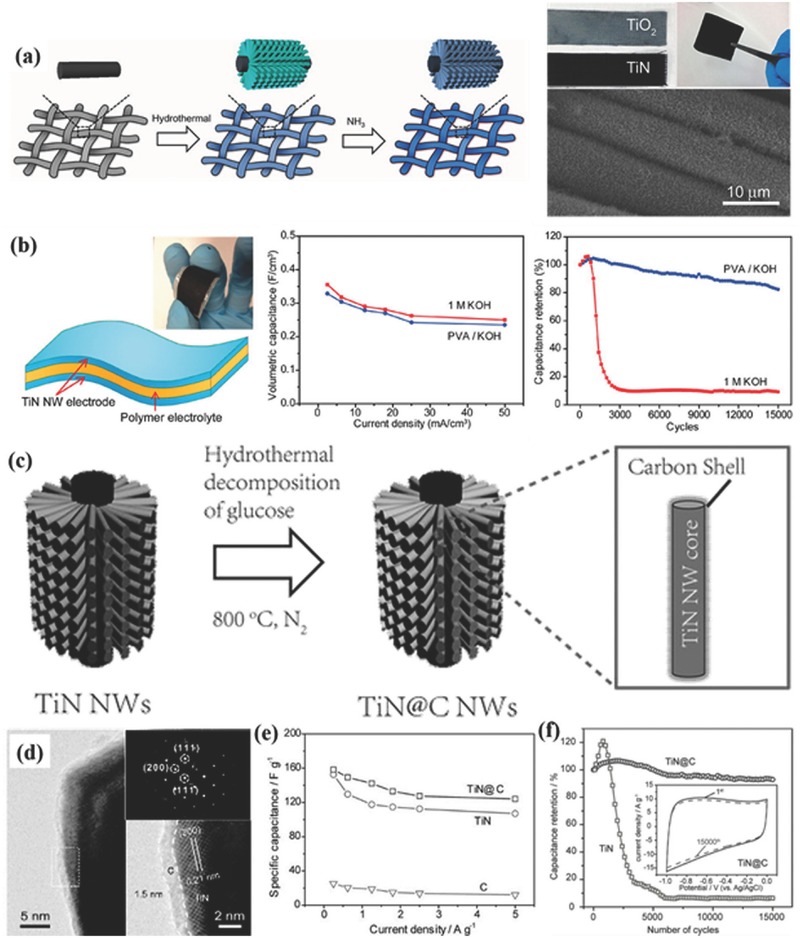
a) Schematic diagram illustrating the two‐step growth process for TiN nanowires on carbon cloth substrate, and photographs of carbon cloth coated with TiO_2_ nanowires and TiN nanowires. b) A schematic diagramof solid‐state TiN nanowires based supercapacitor with PVA/KOH as solid‐state electrolyte and its corresponding volumetric capacitance and cycling stability compared to those with 1 m KOH as electrolyte. Reproduced with permission.^42^ Copyright 2012, American Chemical Society. c) Schematic diagram illustrates the process of depositing protective carbon shell onto TiN nanowires. d) HRTEM images indicating the carbon shell on TiN nanowire. e) The specific capacitance and f) the cycling performance of carbon coated TiN nanowires. Reproduced with permission.^43^

In spite of metal oxides, 1D nanoarrays of metal nitrides and carbide have recently received increasing attention as electrode materials for high‐performance supercapacitors because of their high specific capacitance and especially their excellent electrical conductivity compared to metal oxides.^42^, ^43^, ^44^, ^45^, ^46^, ^47^ Generally, the free‐standing TiN nanowire arrays are grown on different conductive substrates (nickel plate, stainless steel, or carbon fabric) by a template‐free two‐step process as illustrated in **Figure** **3**a.^42^ In details, TiO_2_ nanowire arrays are firstly grown on a substrate via a seed‐assisted hydrothermal method without any template. Second, the as‐prepared TiO_2_ nanowire arrays are thermally annealed in ammonia (NH_3_) at a range of temperatures between 700 and 1000 °C to convert TiO_2_ to TiN. When these template‐freely synthesized TiN nanowire arrays are utilized as supercapacitor electrodes, the free‐standing 1D structure undoubtedly offers a large surface area for surface reactions as well as enables fast intercalation/deintercalation of ions, and at the same time the highly crystalline TiN nanowires have a much higher electrical conductivity than TiO_2_ nanowires to efficiently reduce charge transport resistance, thus both high capacitance and excellent rate capability could be realized simultaneously.^44^ However, metal nitrides suffer from the poor cycling stability, arising from irreversible oxidation reactions in the presence of water and/or oxygen. To solve this problem, the aqueous electrolyte were replaced by the polymer electrolyte to protect the TiN nanowires from electrochemical oxidation and structural degradation, and then solid‐state symmetric supercapacitors based on TiN nanowires were fabricated by sandwiching poly(vinyl alcohol)/KOH based solid‐state electrolyte between two identical TiN nanowires electrodes (Figure 3b).^42^ Subsequently, compared to the device with 1 m KOH as electrolyte, the solid‐state device with KOH/PVA as solid‐state electrolyte had achieved an excellent volumetric specific capacitance of 0.33 F cm^–3^ and an energy density of 0.05 mWh cm^–3^, meanwhile a significantly improved cycling stability that retained 83% of its initial capacitance after 15000 cycles (Figure 3b). Furthermore, inspired by carbon coating stabilized electrodes in Li‐ion batteries,^48^, ^49^ an ultrathin and stable amorphous carbon protective layer was coated onto TiN nanowires surface to suppress the detrimental electrochemical oxidation and structural pulverization of TiN 1D nanoarrays (Figure 3c).^34^, ^43^ As can be seen from Figure 3d, an ultrathin carbon film with the thickness ≈1.5 nm are uniformly coated on the surface of TiN nanowires. The electrochemical experiment results proved that the carbon shell could effectively suppress the electrochemical oxidation reaction of TiN like the polymer electrolytes and thus help to retain their arrayed structure, and at the same time it does not bring negative affect on the charge storage performance of TiN (Figure 3e). Consequently, a remarkable 91.3% retention of the initial capacitance had been achieved in aqueous electrolyte after 15000 cycles (Figure 3f), which is even higher than the values obtained in solid‐state devices.^43^ In contrast to TiN, titanium carbide (TiC) also has high electrical conductivity, but it has much better chemical and thermal stability.^50^ Xia et al. therefore prepared nanotubular TiC fibers to take advantages of TiC for supercapacitors.^51^ Remarkably, the symmetric supercapacitor with nanotubular TiC fibers‐based electrodes exhibited excellent EDLC performance in terms of high capacitance and high rate capability attributing to the high electrical conductivity as well as the nanotubular structure of TiC. More importantly, the full device could work in a wide temperature range from –15 to 65 °C with an increased capacitance and an excellent cycling stability for at least 50000 cycles. It is noted that the working temperature range of TiC‐based supercapacitors might be further increased when optimizing the polymer electrolytes.

**Figure 4:**
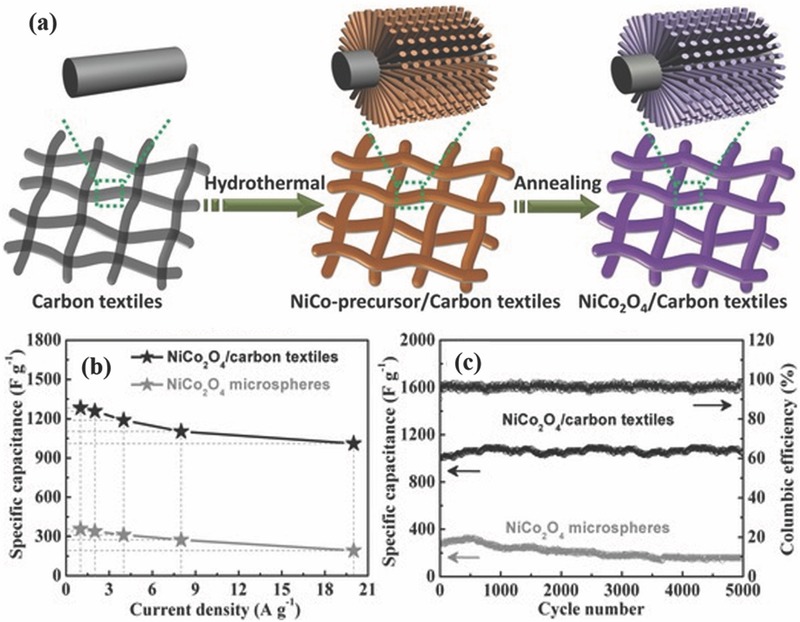
a) Schematic illustration of synthesizing NiCo_2_O_4_ nanowires on carbon textiles. b) SEM images of NiCo_2_O_4_ nanowires on carbon textiles. A comparison of the (b) specific capacitance and (c) cycling performance between NiCo_2_O_4_ nanowires/carbon textiles electrode and NiCo_2_O_4_ microspheres. Reproduced with permission.^54^

Recently, binary metal oxides with a general formula of AB_2_O_4_ have attracted significant attention as suitable electroactive materials for electrochemical energy storage, mainly because they possess multiple oxidation states that enable multiple redox reactions, and thus exhibit a higher supercapacitive performance than any single component metal oxides.^15^, ^52^ Particularly, nickel cobaltite (NiCo_2_O_4_), one of the most typical binary metal oxides for supercapacitors, has been reported to have excellent electrical conductivity and ultrahigh theoretical specific capacitance benefiting from the pseudocapacitive contributions of both the nickel and cobalt ions. Till now, several template‐free methods, including hydrothermal, sol‐gel, microwave, and electrodeposition, etc., have been widely employed for the preparation of binary metal oxides nanostructured electrodes with the geometry of arrayed nanowires, nanorods, nanotubes or nanoneedles on conductive substrate as binder‐free electrodes. Typically, 1D nanoarrays of NiCo_2_O_4_ are synthesized on conductive substrates such as Ni foam, carbon textile, and Ti or stainless steel foil through a hydrothermal reaction together with a post‐annealing treatment (**Figure** **4**a).^53^, ^54^ In details, 1D nanoarrays of NiCo‐precursors were firstly grown on carbon textiles, then they were converted into 1D nanoarrays of NiCo_2_O_4_. NiCo_2_O_4_ nanowires were vertically grown on carbon textiles to form a large‐scale conformal coating. Compared to the conventional binder‐enriched electrode composed of NiCo_2_O_4_ microspheres with obviously increased additional undesirable interparticle resistance, which failed to provide efficient electron transport between electroactive materials and current collector substrate, NiCo_2_O_4_ nanowires/carbon textiles electrode can be directly served as a binder‐free electrode with advanced features: (1) NiCo_2_O_4_ nanowires directly grown on the carbon textiles with robust adhesion ensure intimate contacts and effective electron transport between carbon textiles (as current collector) and each NiCo_2_O_4_ nanowire; (2) large open interspaces between the neighboring nanowires make most of the surfaces of NiCo_2_O_4_ nanowires be easily accessible by the electrolyte, providing effective ion transport pathway. As expected, the NiCo_2_O_4_ nanowires/carbon textiles electrode had exhibited more superior supercapacitor performance than the traditional NiCo_2_O_4_ microsphere electrode with respect to specific capacitance (Figure 4b) and cycling stability (Figure 4c), respectively. Furthermore, with the similar method, the Shen group realized NiCo_2_O_4_ nanowire arrays grown on flexible substrates, including Ni foam, carbon cloth, Ti foil and even polytetrafluoroethylene tape, for constructing flexible supercapacitors.^55^ As a proof of concept, a flexible all‐solid‐state supercapacitors was fabricated with a symmetric configuration by using two NiCo_2_O_4_ nanowire arrays supported on Ni foams as the positive and negative electrodes. The as‐fabricated device had excellent electrochemical performance with a high cell areal capacitance of 161 mF cm^–2^ at 1 mA cm^–2^. Importantly, good electrochemical performance stability over 3000 cycles was obtained even when the device was under harsh mechanical conditions including both twisted and bent states. Besides NiCo_2_O_4_, 1D nanoarrays of other binary metal oxides such as ZnCo_2_O_4_,^56^ NiMo_2_O_4_,^57^ CuCo_2_O_4_,^58^ and (Cu, Ni)O^59^ had been synthesized through different techniques, respectively, on conductive substrates to be studied as binder‐free supercapacitor electrodes.

Additionally, some electroactive materials can be template‐freely electrospun into fibers with diameters ranging from tens of nanometers to several micrometers as self‐supported electrodes for supercapacitors.^60^ The general fabrication process includes electrospinning and/or further annealing. Specially, the electrospun nanofibers of some conductive polymers can be directly used as binder‐free electrodes for supercapacitors without annealing.^61^ When only a polymer as precursor, porous carbon nanofibers are the main product after annealing.^62^ In many cases, some removable additives were added to the precursor solutions in order to tailor the pore geometries of carbon nanofibers. For example, Sun et al. utilized SiO_2_ nanoparticles as additives to synthesize porous carbon nanofibers via electrospinning.^63^ The as‐obtained carbon nanofibers exhibited a periodic distribution of interior holes along the length that is very similar to the structure of bamboos. Such unique structure was found to have excellent mechanical stability under different mechanical deformation conditions, and meanwhile provided high and conductive surface area accessible to the electrolyte. As a result, the as‐prepared bamboo‐like porous carbon fibers were very promising electrode candidate for fabricating flexible supercapacitors without the need of any flexible substrates. Especially, the symmetric device exhibited superior stability with nearly 100% capacitance retention under continuous dynamic operations of forceful bending (90°) and twisting (180°). Nanofibers of pseudocapacitive materials can also be synthesized with electrospinning technique with the precursor solutions consisting of both the supported polymer and the precursors of pseudocapacitive materials. Taking V_2_O_5_ nanofibers as an example, a homogenous solution of poly(vinylpyrolidone) (PVP) and vanadyl acetylacetonate (as precursor of V_2_O_5_) was firstly electrospun into nanofibers, then converted into V_2_O_5_ nanofibers through further thermal annealing.^64^ The annealing conditions were found to have great effect on the microstructures of V_2_O_5_ nanofibers, and then the final electrochemical properties. The optimized specific capacitance for V_2_O_5_ nanofibers was 250 F g^–1^ when the annealing process was conducted at 400 °C. With the similar strategy, nanofibers of some other pseudocapacitive materials such as MnO_2_, α‐Fe_2_O_3_, Co_3_O_4_, and NiO have been synthesized, respectively, and were studied for supercapacitor applications.^65^, ^66^, ^67^, ^68^

Obviously, the key aim in design and fabrication of self‐supported homogeneous nanoelectrodes is to reach high and ion‐accessible specific surface area as much as possible. As regarding 1D nanoarrays, high‐aspect‐ratio ideally leads to increasing both the mass loading and the specific surface area, however, high‐aspect‐ratio 1D nanoarrays tend to agglomerate and collapse (as evidenced in Figure 1b and 1c). The agglomeration will decrease the effective ion‐accessible surface area and meanwhile increase the ion‐transport resistance, consequently imposing adverse effects on supercapacitor performance, especially the rate capability. Therefore, self‐supported homogeneous nanoelectrodes in the form of 2D nanoarrays or 3D nanoporous architectures are potentially more advantageous, but it is noted that not all electroactive materials could be easily fabricated into arrayed 2D or 3D nanostructures.

### 2.2 Self‐Supported Homogeneous 2D Nanoarrays

**Figure 5:**
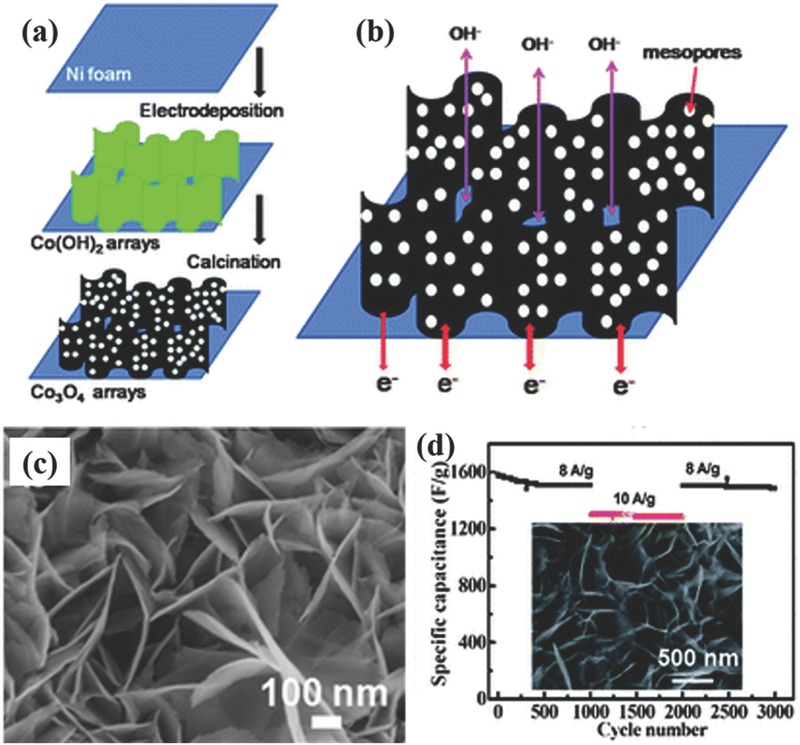
Schematic illustrations of (a) synthesizing process and (b) structural advantages of 2D mesoporous Co_3_O_4_ ultrathin nanosheet arrays on Ni foam. c) SEM image of the Co_3_O_4_ nanosheet nanoarrays. d) Cycling performance of Co_3_O_4_ nanosheet arrays/Ni foam electrode at varying current densities. Reproduced with permission.^71^ Copyright 2012, Royal Society of Chemistry.

2D nanoarrays, such as nanosheets, nanowalls, and nanoflakes have aroused considerable attentions because they also possess the mutual merits with 1D nanostructures as electrode materials for supercapacitors, such as large specific surface area to improve the electrode/electrolyte interfaces, porous feature to increase the amount of electroactive sites, direct pathways for electron transport, and shortened pathways for electrolyte ions diffusion.^69^ In most cases, the 2D nanoarrays are usually synthesized via a template‐free method. Guan et al. prepared porous CoO nanowalls using hydrothermal method on Ni foam.^70^ The CoO nanowalls with a size of 4–5 µm were vertically aligned on the surface of nickel foam and possess a cross‐linked character that many small nanobricks are tightly interconnected with each other, enabling easy access of electrolyte to the entire walls as well as high structural stability. Owing to the highly porous and crystalline structure of CoO nanowalls as well as the direct connection of CoO nanowalls with the macroporous nickel foam, the CoO nanowall arrays exhibited not only high capacitance (≈2.3 F cm^–2^) but also long cycling stability for 5000 cycles. Very similarly, Yuan and co‐workers prepared ultrathin mesoporous Co_3_O_4_ nanosheet arrays supported on Ni foam by a template‐free two‐step process involving electrodeposition of hydroxides followed by a calcination process (**Figure** **5**a).^71^ They also concluded that the open space between the nanosheets and the mesopores existing in the nanosheets can facilitate the fast penetration of the electrolyte to enlarge electroactive surface area for achieving high electrochemical utilization of the electroactive materials (Figure 5b). As can be seen from the SEM image (Figure 5c), the Co_3_O_4_ nanosheets (≈10 nm in thickness) lie aslant or perpendicular to the Ni foam surface, and are interconnected with each other, forming an ordered nanoarrays with a highly open and porous structure. Thus, most of the nanosheet surface is highly accessible by the electrolyte when used as an electrode for supercapacitors. As a result of these advantageous structure features, the specific capacitance of the as‐synthesized ultrathin mesoporous Co_3_O_4_ nanosheet arrays was measured to be as high as 2735 F g^–1^ at a current density of 2 A g^–1^, and had shown excellent cycling stability up to 3000 cycles (Figure 5d). After that, quite a few pseudocapacitive materials had been synthesized into arrayed nanosheet, nanowall, or nanoflake onto conductive substrates though the very similar methods as binder‐free electrodes.^72^, ^73^, ^74^, ^75^, ^76^

Additionally, free‐standing 2D NiCo_2_O_4_ nanosheet arrays have been successfully prepared by different methods and their enhanced electrochemical performance has been well investigated. Recently, Zhang et al. reported the preparation of NiCo_2_O_4_ mesoporous nanosheets using a simple solution method covering various conductive substrates such as Ni foam, Ti foil, stainless steel foil, and flexible graphite paper with robust adhesion as a binder‐ and conductive‐free electrode for supercapacitors.^77^ The Brunauer‐Emmett‐Teller (BET) surface area of these NiCo_2_O_4_ nanosheets was measured to be 112.6 m^2^ g^–1^ with pore size in mesoporous regime (2–5 nm). A reasonably high areal capacitance of 3.51 F cm^–2^ at a current density of 1.8 mA cm^–2^ was subsequently reached. Such high capacitance mainly benefits from the mesoporous features of NiCo_2_O_4_ nanosheets to largely increase the amount of electroactive sites as well as the highly porous film structure formed by the interconnected nanosheets to greatly facilitate transport of electrolyte. In addition, the interconnected nanosheets strengthen the electrode structure to avoid the structure collapse upon cycling, leading to excellent cycling stability. Furthermore, a facile two‐step process, electrodeposition and subsequent calcination, has been developed by the same group for large‐scale growth of ultrathin NiCo_2_O_4_ mesoporous nanosheets on Ni foam.^78^ The as‐prepared NiCo_2_O_4_ mesoporous nanosheets were composed of only 3–6 layers of NiCo_2_O_4_ atomic sheets, and possessed numerous interparticle mesopores of 2–5 nm in size, providing much larger electroactive surface area. Consequently, superior supercapacitor performance was achieved with high capacitance and excellent cycling stability at a larger current densities.

**Figure 6:**
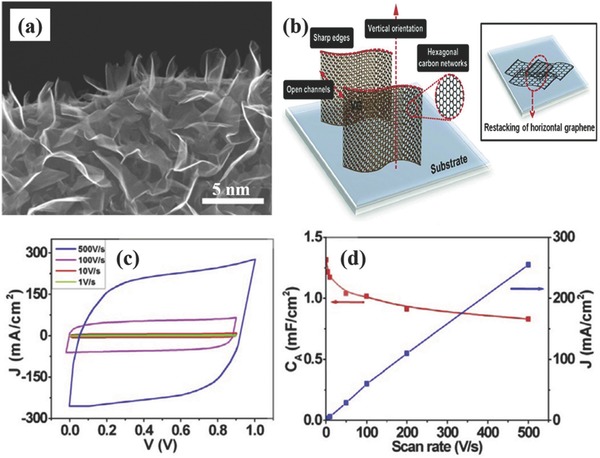
a) Typical SEM image of VG grown on a substrate. Reproduced with permission.^81^ Copyright 2014, American Chemical Society. b) Schematic illustration of VG's structural and morphological features. Inset illustrates the restacking of horizontal graphene nanosheets. Reproduced with permission.^79^ Copyright 2015, Royal Society of Chemistry.c) CV curves of a VG‐based supercapacitor electrode at scan rates of 1–500 V s^–1^ and (d) the corresponding specific capacitance. Reproduced with permission.^82^ Copyright 2014, Elsevier.

Vertically‐oriented graphene (VG), which graphene nanosheets are growth perpendicularly on the conductive substrate surface normally through a plasma‐enhanced chemical vapor deposition (PECVD) process, has many unique morphological and structural features such as non‐agglomerated 3D inter‐networked morphology, controlled inter‐sheet connectivity, as well as long exposed ultra‐thin and reactive graphene edges, making them very attractive for supercapacitor applications.^79^ **Figure** **6**a indicates the typical morphology of VG grown on a substrate. The VG with large and readily accessible surface area is beneficial to achieve a higher EDLC capacitance (Figure 6b). Moreover, it is believed that the dense edge planes of VG can also enhance the charge storage capability because the edge planes have a much larger area‐specific capacitance than the basal plane surface (Figure 6b).^80^ When VG grown on a conductive substrate is applied as binder‐free electrodes, a significant feature of VG‐based supercapacitors is the ultrafast dynamic response in a high frequency mode, attributing to the vertical orientation and open inter‐sheet channels to favor ion migration among electrodes as well as the intrinsic good in‐plane electrical conductivity of graphene to facilitate charge transport within active materials. For example, the VG‐based EDLC was reported to exhibit an impedance phase angle of –84° at 120 Hz, which is very close to the ideal capacitive behavior (–90°) and thus is very desirable for the 120 Hz alternative current (ac) line‐filtering application.^80^, ^81^ What is more, the VG‐based EDLC presented much higher volumetric energy density than the commercial Al electrolytic capacitor owning an impendence phase angle of –83° at 120 Hz, benefiting for device minimization of ac line‐filtering system. Furthermore, the VG‐based EDLCs have been reported to have ultrahigh rate capability, which the shape of cyclic voltammetry (CV) curves maintains quasi‐rectangle even at a scan rate as high as 500 V s^–1^ (Figure 6c) with the specific capacitance maintaining at 0.83 mF cm^–2^ (Figure 6d).^82^ Considering that the charge storage of VG is based mainly on the EDLC mechanism, to further increase the EDLC capacitance but maintain high rate capability, Seo et al. had directly grown carbon nanotubes (CNTs) onto VG to form a 3D hybrid structure with increasing specific surface area.^83^ The synergistic combination of VG and CNTs enabled to achieve an increased surface area, leading to a high specific capacitance of 278 F g^–1^ at 10 mV s^–1^ and the capacitance retention of >99% after 8000 cycles.

### 2.3 Self‐Supported 3D Nanoporous Homogeneous Nanoelectrodes

In spite of 1D and 2D homogeneous nanoelectrodes, some electroactive materials like graphene have been able to be synthesized into self‐supported 3D nanoporous or macroporous structures.^84^, ^85^ As known, the restacking and aggregation of graphene nanosheets due to the π–π stacking interaction and van der Waals attraction among their basal planes lead to significant loss of their surface area and also make it difficult for electrolyte ions to access the interspace among the densely packed graphene sheets.^79^ One of the efficient ways to solve this problem is to assemble 2D graphene sheets into 3D porous graphene framework, not only preventing the sheets from restacking, but also enabling easy access of electrolyte to the electrode surface. Besides VG that 2D graphene nanosheets are vertically growth on conductive substrate, 3D porous graphene materials have emerged as promising candidates for supercapacitors due to their excellent electrical conductivity and large surface area.^86^, ^87^, ^88^ A number of 3D graphene materials with novel 3D structures have been developed in recent years with a number of synthetic methods based on the strategies of either template‐free or template‐assisted method.

#### 2.3.1 Template‐Free Method

As a typical example of 3D graphene materials, laser‐scribed graphene (LSG) was produced from the laser scribing of graphene oxide (GO) films drop‐casted onto a flexible substrate.^89^ After irradiation of the GO film with an infrared laser, the initially stacked GO nanosheets were converted into well‐exfoliated LSG sheets. The as‐prepared LSG has three key advantages for supercapacitor applications. First, the LSG has large and accessible specific surface area accounting for high areal and volumetric capacitance. Second, the LSG has an open network to prevent the agglomeration of graphene nanosheets, and meanwhile to facilitate ion diffusion in LSG electrodes. In comparison with chemically converted graphene (CCG) films in which the CCG sheets are well connected together in a layered structure to form the CCG electrodes, the LSG electrodes with well‐defined porous structure and large accessible surface exhibited a rapid frequency response of 23 ms, while a slow frequency response of ≈5 s for CCG electrodes due to the reduced porosity and limited accessibility to electrolyte ions.^90^ Last, the LSG possesses excellent electric conductivity of 1738 S m^–1^ benefiting to achieving high power. It has been proved that the LSG electrode has an excellent EDLC behavior with a nearly rectangular CV shape at a scan rate of 1000 mV s^–1^. Attributing to these advanced features, the symmetric LSG supercapacitor reached an energy density of up to 1.36 mWh cm^–3^ (approximately two times higher than that of the AC‐EC) and a power density of ≈20 W cm^–3^ (20 times higher than that of the AC‐EC and three‐orders of magnitude higher than that of the 500‐mAh thin‐film lithium battery).^89^

**Figure 7:**
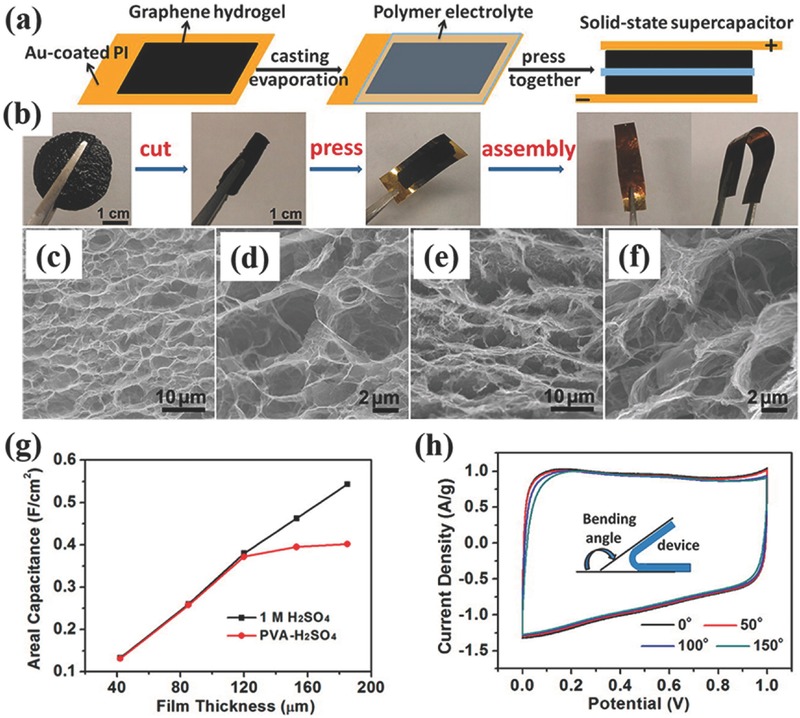
a) Schematic diagram and b) photographs of fabricating flexible solid‐state supercapacitors based on graphene hydrogels. SEM images of the interior microstructure changes of the graphene hydrogel (c–d) before and (e–f) after pressing. g) Thickness dependent areal capacitance of a graphene hydrogel film. h) CV curves of the flexible solid‐state device at 10 mV s^–1^ for different bending angles. Reproduced with permission.^92^ Copyright 2013, American Chemical Society.

Besides LSG, graphene hydrogels/aerogels are another kind of 3D graphene materials consisting of interconnected graphene networks with unique characteristics including a large pore volume, high specific surface area and tunable porosity.^91^ The general way for producing graphene hydrogels/aerogels is to replace the water entrapped in hydrogels with air by means of hydrothermal method, freezing drying or supercritical drying, yielding 3D graphene networks with pore sizes ranging from submicrometers to several micrometers. For instance, Xu et al. synthesized free‐standing 3D graphene hydrogel by a hydrothermal reduction method as electrodes for fabricating solid‐state flexible supercapacitors.^92^ The process for fabricating solid‐state flexible supercapacitors includes cutting, pressing, polymer electrolyte casting, and assembling (**Figure** **7**a and 7b). The as‐prepared 3D graphene hydrogels possessed an interconnected porous network with most of its pores having a size of several micrometers as can be seen from Figure 7c and 7d. Importantly, the 3D continuous porous network was well maintained upon physical pressing, e.g., the graphene hydrogel was compressed from a thickness of ≈3 mm to ≈120 µm during assembling supercapacitor device (Figure 7e and 7f). Interestingly, the conductivity of graphene hydrogels was also increased from 7.8 to 192 S m^–1^ after compressing. As shown in Figure 7g, the final solid‐state flexible device shows film thickness dependent areal capacitance as well as an extraordinary mechanical flexibility (Figure 7h). A largest areal specific capacitance of 372 mF cm^–2^ was obtained at a film thickness of only 120 µm. In most supercapacitor device fabrication based on graphene hydrogels, it is worth emphasizing that the graphene hydrogels impregnated with electrolyte was physically pressed onto a conductive substrate to form a thin film electrode.^93^, ^94^, ^95^ Since the graphene hydrogels are just physically attached onto the current collector, the imperfections of the contact surface between the current collector and graphene hydrogels might lead to some energy loss as the result of the electrical contact resistance.^96^, ^97^ Therefore, directly synthesizing graphene hydrogels onto a conductive substrate might create continuous contact through chemical means or nanostructuring the conductive substrate, and then help to reduce the ohmic losses, resulting in further enhanced energy density of the assembled supercapacitor devices.

#### 2.3.2 Template‐Assisted Method

**Figure 8:**
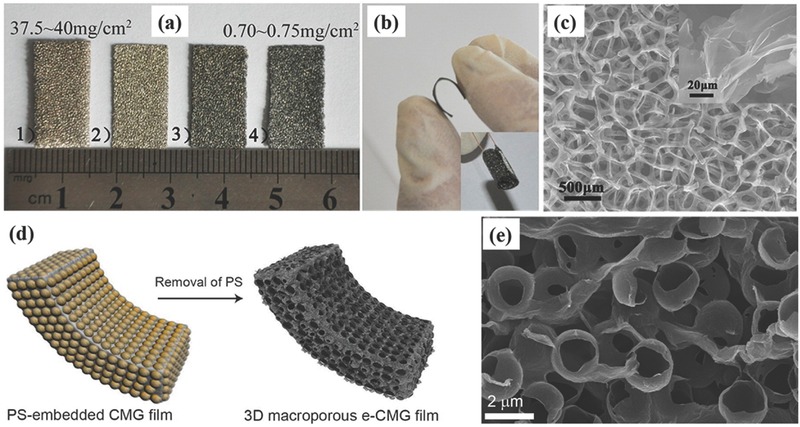
(a) Photographs of (1–2) Ni foams and graphene‐coated Ni foams (3) before and (4) after removal of the Ni networks. b) Photograph and c) SEM image of a freestanding and flexible 3D graphene network. Reproduced with permission.^98^ Copyright 2013, American Chemical Society. d) Schematic illustrating the fabrication of 3D macroporous graphene films through an embossing process using PS templates, and (e) cross‐sectional SEM images of the as‐obtained 3D macroporous graphene films. Reproduced with permission.^99^ Copyright 2012, American Chemical Society.

Compared with template‐free methods, template‐assisted methods endow 3D graphene materials with much more controlled morphologies by using predesigned 3D sacrificial templates. For example, with Ni foam as a sacrificial scaffold and also catalyst, He et al. prepared freestanding flexible 3D graphene networks through CVD process followed by Ni etching.^98^ **Figure** **8**a shows the color change of Ni foam before and after the deposition of graphene, indicating the successful growth of graphene on Ni foam. After etching the Ni foam, the obtained flexible 3D graphene networks exhibits excellent flexibility (Figure 8b) and importantly, it resembles the macroscopic morphology of initial Ni foam and integrated the graphene sheets into a macroscopic monolith (Figure 8c). And Choi et al. employed a conventional replicating and embossing technique to fabricate 3D macroporous graphene films by using multi‐layer polystyrene (PS) colloidal spheres as sacrificial templates as schematically illustrated in Figure 8d.^99^ Figure 8e indicates the interconnected nature of the multilayered graphene walls in the assembled 3D structure. And it has been revealed that all these obtained 3D graphene networks show large specific surface area, high conductivity, and excellent flexibility, thus are promising for applications in flexible supercapacitors. It is also found that these 3D graphene networks could be an ideal supporter and current collector for depositing pseudocapacitive materials towards constructing heterogeneous nanoelectrodes. Not only 3D graphene networks prepared by template‐assisted methods, with monolayer PS colloidal spheres as template, Zhong et al. prepared monolayer TiC hollow sphere arrays. The porous hollow structure and high conductivity of TiC hollow sphere arrays contributed to achieve superior EDLC performance. More impressively, the symmetric supercapacitors with an organic electrolyte (1 m EMIMBF_4_ as the electrolyte) exhibited excellent stability operating up to 3 V at high temperature (65 °C), which should be attributed to the structural robustness of TiC hollow sphere and the good anti‐oxidation capability of TiC itself.^100^

## 3 Self‐Supported Heterogeneous Nanoelectrodes

Many pseudocapacitive materials like transition metal oxides/hydroxides can store high‐density energy via fast surface redox reactions, however, their well‐known poor conductivity causes remarkable reductions in the energy storage of most pseudocapacitors at high power delivery (fast charge/discharge rates). Construction of core‐shell heterostructures with the combination of two or more different materials has been proved as a promising strategy to boost the electrochemical performance of transition‐metal oxides/hydroxides.^101^ Unlike homogeneous nanoelectrodes, the self‐supported heterogeneous nanoelectrodes generally adopt a configuration of current collector core – active material shell, which a very thin layer of electroactive materials is conformally coated onto a self‐supported nanostructured current collector/supporter. Such configuration could achieve very high specific capacitance, however, the mass loading of electroactive materials is very low. The materials of current collectors are usually highly‐conductive metals, but there are also some cases that both the core and the shell are transition metal oxides and in such cases the transition metal oxide as core are normally have better electrical conductivity than the shell ones and can be easily prepared into self‐supported nanoarrays. The configuration of core‐shell heterostructures are intended to ensure a short electron transport length for all the pseudocapacitive materials in order to reach the full potential of pseudocapacitive materials for electrochemical energy storage. As a result, the energy storage capability would depend largely on the configuration of core‐shell heterostructure except the charge storage material itself. To design an ideal heterogeneous nanoelectrodes, three key criteria should be satisfied, e.g., a high specific surface area leading to an improved specific capacitance, a high electronic and ionic conductivity helping to enhance the rate capability, and an excellent mechanical and chemical stability to boost the cycle stability.^14^ In most cases, the self‐supported core‐shell heterostructures are hybrid 1D nanoarrays or 2D nanoarrays of synthesized on different conductive substrates (i.e., foil, wire, and foam, etc.). Moreover, some 3D nanoporous core‐shell heterostructures also have been reported for supercapacitor applications.

### 3.1 Self‐Supported Heterogeneous 1D Nanoarrays

Typically, 1D heterogeneous nanoelectrodes are in the form of core‐shell arrays prepared by conformally coating electroactive materials on a 1D nanowire/nanotube arrays current collector grown on a conductive substrate.^102^ As 1D nanoarrays current collectors benefit for the accessible diffusion of electrolytes and faster charge transport, the core‐shell structure could boost the electrochemical energy storage.^10^, ^32^, ^103^, ^104^, ^105^, ^106^ Arrayed 1D nanostructures (e.g. nanorod, nanowire, nanotube, etc.) of metals and other conductive materials (for example, ZnO, TiO_2_, SnO_2_, and TiN) have been extensively studied as current collectors for supercapacitors owing to their inherent advantages in terms of high electrical conductivity, high surface area and shortened electronic/ion transport pathways.^107^, ^108^, ^109^, ^110^ Depending on whether a template is required for producing 1D nanoarrays current collectors, the reported synthetic methods for fabricating 1D heterogeneous nanoelectrodes include template‐assisted and template‐free method. The following gives some representative examples.

#### 3.1.1 Template‐Assisted Method

**Figure 9:**
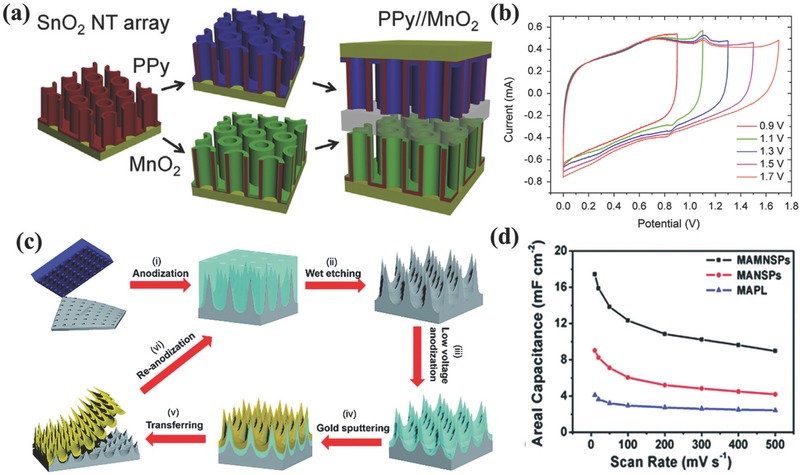
a) Asymmetric supercapacitor with SnO_2_@PPy nanotube arrays as negative electrode and SnO_2_@MnO_2_ nanotube arrays as positive electrode. b) The working potentials for SnO_2_@MnO_2_//SnO_2_@PPy asymmetric supercapacitor. Reproduced with permission.^117^ Copyright 2014, Elsevier. c) Schematic illustration showing the fabrication process of free‐standing Au nanospikes film. d) Areal capacitance comparison of MAMNSP, MANSP, MAPL electrodes. Reproduced with permission.^120^ Copyright 2015, Royal Society of Chemistry.

Template‐assisted method is simple and efficient for preparing 1D nanowire/nanotube arrays. By initially controlling the structural parameters of template, the template grown nanowires/nanotubes provide for good control of spacing between individual nanowires/nanotubes for the electrode/electrolyte layers and improve surface area. For example, by utilizing AAO templates along with different fabrication techniques, oriented nanowire/nanotube arrays of certain conductive materials have been easily produced as current collectors, and they are further employed to construct 1D heterogeneous nanoelectrodes, such as Ni@MnO_2_,^111^ Ni@NiO,^112^, ^113^ Pt@MnO_2_,^114^ TiN@MnO_2_,^115^ SnO_2_@MnO_2_,^116^ SnO_2_@PPy,^117^ and CNT@MnO_2_,^118^ etc. What these aforementioned 1D heterogeneous nanoelectrodes in common is that the AAO template‐directed 1D nanoarrays current collectors provide direct pathways for fast electron transport and straight channels for ion conduction, meanwhile the large surface area to achieve high specific pseudocapacitance together with a very thin layer of pseudocapacitive materials. As an example shown in **Figure** **9**a, the asymmetric supercapacitor using SnO_2_@PPy core‐shell nanotube arrays as negative electrode and SnO_2_@MnO_2_ core‐shell nanotube arrays as positive electrode achieved remarkable energy storage capability with an enlarged potential window (Figure 9b).^117^ In addition to nanowire/nanotube arrays, metal coated nanocone arrays of supercapacitive‐inert polymers have been prepared based on AAO template as 1D nanostructured current collectors for flexible supercapacitors.^119^ Qiu et al. demonstrated the synthesis of Perfluoropolyether (PFPE)/Au@MnO_2_ core‐shell nanocone arrays for supercapacitor application.^119^ PFPE nanocones were fabricated by AAO‐assisted polymer contact printing technique, then Au and MnO_2_ were subsequently deposited forming PFPE/Au@MnO_2_ core‐shell nanocone arrays. This nanoelectrode had achieved a specific mass (areal) capacitance of 840.3 F g^–1^ (88.2 mF cm^–2^) at a current density of 2 A g^–1^. Additionally, Al nanospike arrays were found to be generated during the preparation of AAO template from Al foil, and similar with nanowire/nanotube arrays, the Al nanospike arrays are not only directly used as current collectors but also capable to be utilized as sacrificial template to produce 1D heterogeneous nanoelectrodes for supercapacitors.^120^, ^121^ Gao et al. have fabricated an ultrathin, freestanding gold (Au) nanospikes film coated with MnO_2_ as supercapacitor electrodes.^120^ The fabrication of Au nanospikes is illustrated in Figure 9c. The anodization of nanoimprinted Al foil results in the formation the Al nanospikes under the layer of aluminum oxide, and this Al nanospikes was further anodized into aluminum oxide nanospikes with low anodization potential after removing the top aluminum oxide layer. With this aluminum oxide nanospikes as the starting template, an Au nanospikes was further fabricated after Au sputtering and the following removal of aluminum oxide nanospikes. To form heterogeneous nanoelectrodes, a thin layer of MnO_2_ is finally deposited on the gold nanospikes. In this heterogeneous nanoelectrode, the Au nanospikes increase the effective electrode surface area, facilitate electrolyte permeation, and shorten the electron pathway in the electroactive materials. As can be seen from Figure 9d, these unique merits lead to a considerable enhancement in electrochemical performance, which is 1.9 and 4.26 times higher capacitance as compared to MnO_2_/Au nanospike (MANSP) and MnO_2_/planar (MAPL) electrodes, respectively.

**Figure 10:**
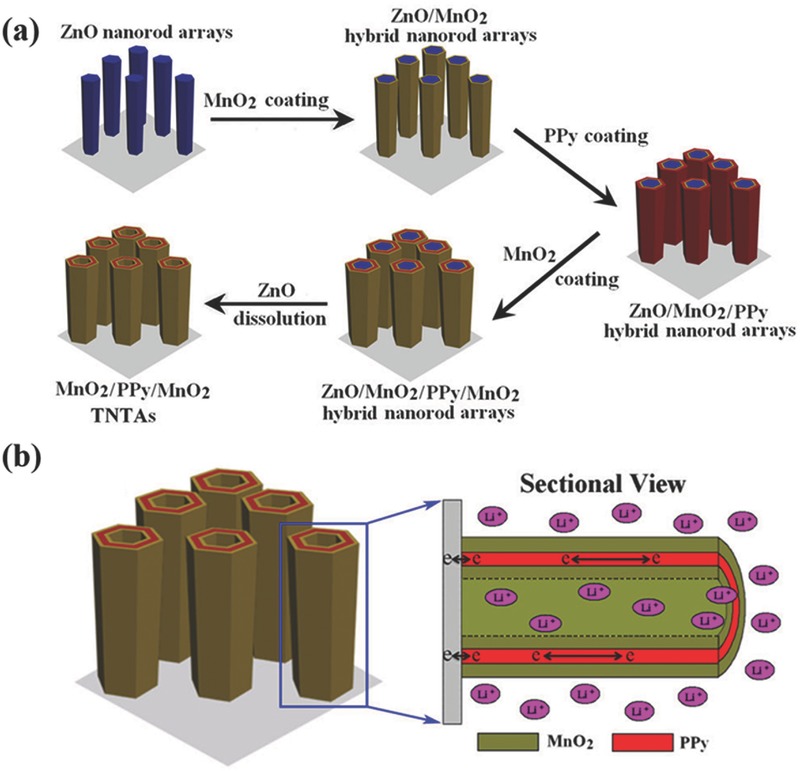
a) Schematic illustration for synthesizing MnO_2_/PPy/MnO_2_ sandwich‐structured nanotube arrays. b) The nanotubular architectures with triple layered structures facilitate both ion and electron transport for rapid charge/discharge processes. Reproduced with permission.^131^ Copyright 2013, Nature Publishing Group.

Recently, template‐freely synthesized ZnO nanowires/nanorods have drawn increasing attentions for supercapacitors and have demonstrated not only as good scaffolds to support active materials due to their relatively good electrical conductivity and large specific surface area,^122^, ^123^ but most importantly as templates to produce novel 1D heterogeneous nanoelectrodes because ZnO can be easily chemically removed as already mentioned above.^124^, ^125^, ^126^, ^127^, ^128^ With ZnO nanorod arrays employed as nanostructured current collectors, Yang et al. fabricated hydrogenated single‐crystal ZnO@amorphous ZnO‐doped MnO_2_ core‐shell nanowires on carbon cloth as supercapacitor electrodes.^122^ In this heterostructure, the aligned and ordered ZnO nanowires with greatly improved electrical conductivity after hydrogenation directly serve as nanostructured current collectors, and finally the electrodes had shown a areal capacitance of 138.7 mF cm^–2^ (specific capacitance of 1260.9 F g^–1^). Additionally, with ZnO nanorods directly grown on conductive substrates as templates and attributing the easy removal of ZnO nanorods, some unique 1D heterogeneous nanoelectrodes had been synthesized mainly in the form of heterogeneous nanotube arrays.^124^, ^125^, ^126^, ^127^, ^128^, ^129^, ^130^, ^131^, ^132^ For instance, starting with template‐freely grown ZnO nanorod arrays as the sacrificial template, Ni were deposited on the surface of ZnO nanorod arrays, followed by dissolving of the ZnO templates to generate Ni nanotube arrays.^127^ Evidently, the Ni nanotube arrays could provide fast electron transport, while the open‐framework nanotube arrays act as the ion reservoir and offer short ion diffusion pathways to accelerate the ion transport. After electropolymerizing highly porous perchlorate‐doped polypyrrole (PPy) layer on Ni nanotube arrays, the obtained Ni nanotubes@PPy electrode were found to efficiently work as both negative and positive electrodes with largely widened and stable operating potential windows. More specifically, the Li group had developed a novel ZnO nanorod template‐assisted electrodeposition route to fabricate unique sandwich‐structured nanotube arrays as supercapacitor electrodes.^130^, ^131^ They designed multi‐walled MnO_2_/PPy/MnO_2_ nanotube arrays with ZnO nanorod as the starting template by multi‐step electrochemical deposition as schematically illustrated in **Figure** **10**a, including (1) growth of ZnO nanorod arrays on substrate; (2) electrodeposition of MnO_2_ on ZnO; (3) electrodeposition of PPy on MnO_2_; (4) electrodeposition of second layer MnO_2_ on PPy; (5) dissolution of ZnO nanorods. In this sandwich‐structured nanotube arrays, the inner and outer surfaces of one conductive nanotube (PPy) were both coated with electroactive materials (MnO_2_). In such configuration (Figure 10b), the middle conductive nanotube provide efficient electron pathways for charge storage and delivery, the hollow nanostructures facilitate the ion transport to enable fast, reversible Faradaic reactions, and the double shells offer larger specific surface area to enhance the utilization of active materials for electrochemical energy storage. Owing to these advanced features, such nanoelectrodes exhibited superior supercapacitive performance with excellent long‐term cycle stability and high energy and power densities.

#### 3.1.2 Template‐Free Method

1D heterogeneous nanoelectrodes can also be fabricated through template‐free methods. Besides producing homogeneous nanoelectrodes, electrospinning is also capable to fabricate heterogeneous nanoelectrodes in the form of nanofibers when the initial electrospinning solution consisting of precursors of two different electroactive materials. The as‐obtained heterogeneous nanofibers can be in multi‐stacked morphology with high porosity to provide large ion‐accessible surface area.^133^ Moreover, Zhou et al. fabricated TiN‐VN core‐shell nanofibers via coaxial electrospinning technique assisted with post annealing.^134^ In the TiN‐VN core‐shell nanofibers, the core TiN with excellent electrical conductivity ensures high rate capability, whereas the shell VN offers high specific capacitance. Moreover, 1D nanoarrays of some metals can also be fabricated via a simply template‐free method as current collectors for further fabricating heterogeneous nanoelectrodes. For example, Xu et al. template‐freely prepared ultra‐long Ni nanowire arrays through a modified magnetic‐field‐driven selective deposition growth process.^135^ The as‐prepared Ni nanowires have the diameter ranging from 120 to 170 nm with aspect ratio of over 8000, and they were employed as the electrode scaffold for MnO_2_ and PPy to construct an aqueous asymmetric micro‐supercapacitor. It has been proved that a higher specific capacitance of pseudocapacitive materials can only be achieved via a thinner layer. Eventually, Ni nanowires with such higher aspect ratio can provide much more nickel/pseudocapacitive materials active sites and thus greatly reduce the thickness of pseudocapacitive materials under the same mass loading, resulting in a superior electrode capacitance. However, the nanowire arrays with high aspect ratio are known to easily agglomerate with the formation of nanowire clusters, leading to significant rate performance degradation mainly because of the block of electrolyte ions flow.

**Figure 11:**
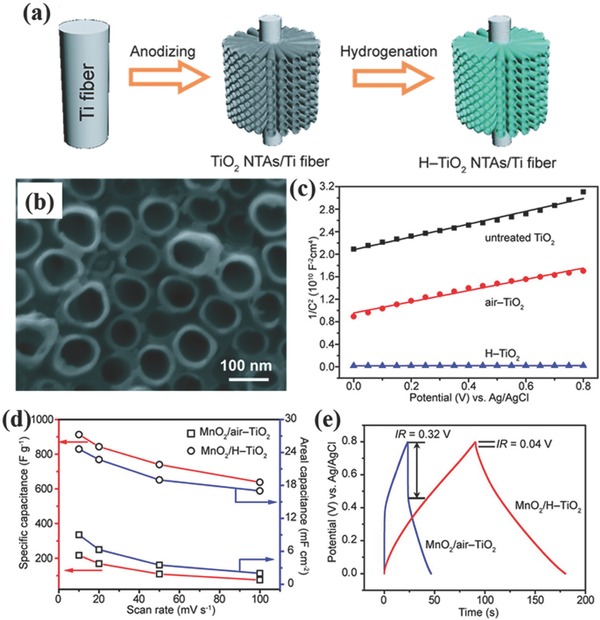
a) Schematically illustrating the fabrication of H‐TiO_2_ nanotube arrays. b) SEM image of H‐TiO_2_ nanotube arrays. c) Mott‐Schottky plots of the untreated TiO_2_, air‐TiO_2_, and H‐TiO_2_ NTAs, indicating a significantly enhanced carrier density in H‐TiO_2_ nanotube. d) Specific capacitances and areal capacitances of the MnO_2_/H‐TiO_2_ and MnO_2_/air‐TiO_2_ composites measured as a function of scan rate, and (e) their galvanostatic charge/discharge curves. Reproduced with permission.^144^ Copyright 2012, American Chemical Society.

Self‐organized vertically‐aligned TiO_2_ nanotube arrays template‐freely prepared by anodic oxidation of Ti substrates are also very attractive scaffold for fabricating 1D heterogeneous nanoelectrodes, attributing to the highly oriented tubular structure offering an extremely large and solvated ion accessible surface area. Several pseudocapacitive materials like RuO_2_,^136^ MnO_2_,^137^, ^138^, ^139^ MoO_3_,^140^ polythiophene,^141^, ^142^ and polyaniline^143^ have been deposited into TiO_2_ nanotubes and have achieved impressive supercapacitive performance. However, the electrical conductivity of TiO_2_ nanotubes is still not as high as enough to functionalize as promising current collectors. Therefore, several strategies had been applied to improve the electrical conductivity of TiO_2_ nanotubes as current collectors, including hydrogenation,^144^, ^145^ cathodic polarization,^146^, ^147^, ^148^ and nitridation.^149^, ^150^, ^151^ Lu et al. reported the hydrogenated TiO_2_ nanotube arrays obtained by calcination of anodized TiO_2_ nanotube arrays grown on Ti wires in hydrogen atmosphere (**Figure** **11**a).^144^ As can be seen from Figure 11b, the H‐TiO_2_ nanotubes well keeps the structure features as the original TiO_2_ nanotubes after annealing. Compared to untreated TiO_2_ nanotubes, hydrogenation leads to a 3 orders of magnitude enhancement in carrier density in TiO_2_ nanotubes, and thus resulting in dramatically increased conductivity (Figure 11c). When hydrogenated TiO_2_ nanotube arrays using as current collector for MnO_2_ coating, the H‐TiO_2_@MnO_2_ heterogeneous nanoelectrodes had shown a remarkable specific capacitance of 912 F g^–1^ at a scan rate of 10 mV s^–1^ (Figure 11d), which was 4 times higher than that of heterogeneous nanoelectrodes with MnO_2_ coated air‐treated TiO_2_ nanotube arrays, and the IR drop of the discharge curves of MnO_2_/H‐TiO_2_ is much smaller than that of MnO_2_/air‐TiO_2_ (Figure 11e). Furthermore, when calcining the anodized TiO_2_ nanotube arrays under ammonia, TiO_2_ nanotubes will be in situ transformed into highly conductive TiN nanotube arrays with much higher conductivity. Attributing to the large surface area, the fast electron transport framework, and the high electric conductivity of TiN nanotubes, the heterogeneous TiN/Ni_x_Co_2x_(OH)_6x_ nanoelectrode exhibited superior pseudocapacitive performance with a high specific capacitance of 2543 F g^–1^ at a scan rate of 5 mV s^–1^, remarkable rate performance of 660 F g^–1^ even at a scan rate of 500 mV s^–1^, and promising cycle performance (about 6.25% capacitance loss for 5000 cycles).^150^

**Figure 12:**
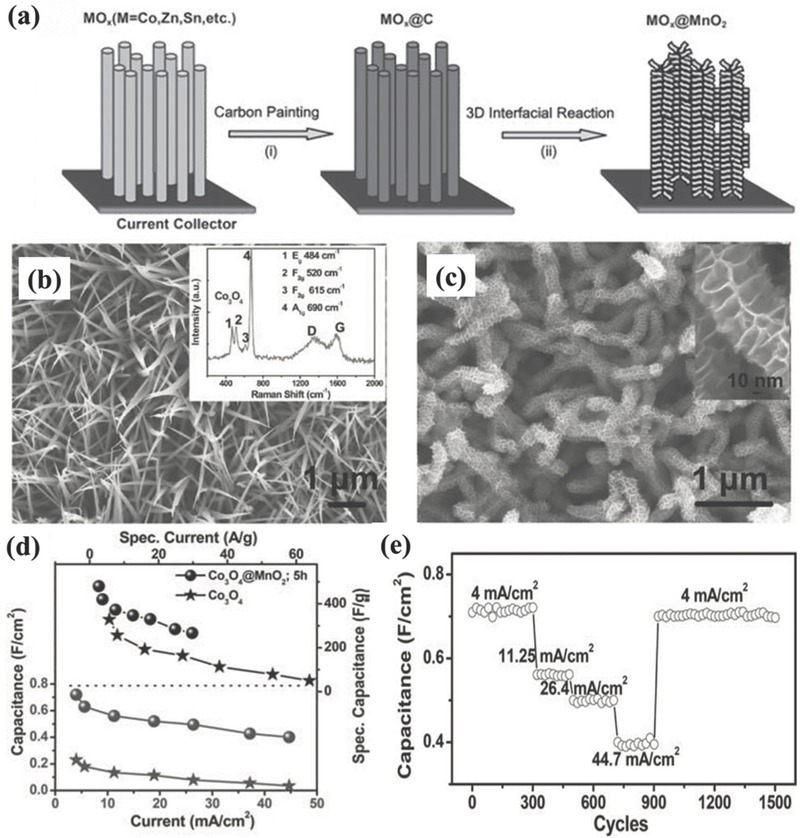
a) Schematic illustrating the fabrication processes of the Co_3_O_4_@MnO_2_ nanoarrays on a stainless steel substrate. SEM image of (b) Co_3_O_4_ and (c) Co_3_O_4_@MnO_2_ nanoarrays, respectively. d) Areal and specific capacitance comparison of Co_3_O_4_ and Co_3_O_4_@MnO_2_ nanoarrays. e) Cycling stability of Co_3_O_4_@MnO_2_ nanoarrays at progressively varied current density. Reproduced with permission.^157^

Moreover, by utilizing nanowire arrays of some transition metal oxides/sulfides synthesized on conductive substrates through template‐free methods (e.g., hydrothermal method and chemical vapor deposition), there is another way to construct hybrid metal oxide core‐shell nanowire arrays as 1D heterogeneous nanoelectrodes for supercapacitors.^152^, ^153^, ^154^, ^155^, ^156^ Such nanoelectrode architecture has following merits.^157^ First, both the core and shell materials are good pseudocapacitive materials. Typically, self‐supported nanowire arrays of metal oxides/sulfides (e.g., Co_3_O_4_, SnO_2_, CoO, CoS, WO_3_, and Fe_2_O_3_) are usually the core, while other metal oxides/hydroxides (e.g., NiO, MnO_2_, Ni(OH)_2_, Co(OH)_2_, CoMoO_4_) with high pseudocapacitance are employed as the shell materials. Both the core and shell materials have redox reactions, thus both contributing to the electrochemical energy storage. Second, the core‐shell heterogeneous nanoarrays can provide many advantages such as enlarged contact surface area between electrode and electrolyte and a short pathway for ion diffusion, and give rise to synergetic effect of the core and shell pseudocapacitive materials. By combining these unique properties, improved performance has therefore been realized in such electrodes. Taking Co_3_O_4_ as an example, hydrothermally synthesized Co_3_O_4_ nanowire arrays (**Figure** **12**a) on conductive substrates served as both the backbone and conductive connection for pseudocapacitive materials like MnO_2_,^157^, ^158^ NiO,^159^ Co(OH)_2_,^160^ CoMoO_4_,^161^ polypyrrole,^162^ etc. As shown in Figure 12b, Co_3_O_4_@MnO_2_ core‐shell nanowire arrays are densely grown on a stainless steel substrate, in which MnO_2_ is conformally coated on Co_3_O_4_ nanowires by the reaction between KMnO_4_ and the pre‐deposited amorphous carbon on Co_3_O_4_ nanowires (Figure 12c). It has been found that the Co_3_O_4_@MnO_2_ core‐shell nanowire arrays exhibit a 4 to 10‐fold increase in areal capacitance with respect to pristine Co_3_O_4_ nanoarrays (Figure 12d) and meanwhile have good cycle performance with only 2.7% capacitance loss after 5000 cycles.^157^ However, still suffering from the lower conductivity of these metal oxides as the core, the rate capability of these heterogeneous nanoelectrodes are unsatisfied, especially at high charge‐discharge rate. As can be seen from Figure 12e, when the current density increasing from 4 to 44.7 mA cm^–2^, the areal capacitance of the heterogeneous nanowire array was only 56% retention of that measured at 4 mA cm^–2^.^157^

To overcome this limitation, some binary metal oxides/sulfides like NiCo_2_O_4_,^163^, ^164^, ^165^, ^166^, ^167^, ^168^, ^169^ NiCo_2_S_4_,^170^, ^171^ CoMoO_4_ 172 with higher conductivity than metal oxides had been adopted to replace these above metal oxides as the pivotal core component for the heterogeneous design. As the above mentioned metal oxides, these binary metal oxides/sulfides are template‐freely synthesized into self‐supported 1D nanoarrays on conductive substrates. Especially, NiCo_2_O_4_ is of significant interest because it possesses not only better electrochemical activity due to the contributions from both the nickel and cobalt ions, but importantly higher electrical conductivity (62 S cm^–1^) at least two orders of magnitude higher than pure Co_3_O_4_ and NiO (10^–3^ – 10^–2^ S cm^–1^).^173^ When constructing heterogeneous nanoelectrodes with 1D nanoarrays of NiCo_2_O_4_ as the core, the NiCo_2_O_4_ nanoarrays in a heterogeneous electrode serve as the multifunctional support for anchoring other electroactive materials. Firstly, the NiCo_2_O_4_ nanoarrays provide the high surface area for the growth of another electroactive nanomaterials, resulting in the increasing ion‐accessible surface area for charge storage. Secondly, both NiCo_2_O_4_ and the anchored electroactive are involved in the redox reactions to contribute to the charge storage capability of the electrode, allowing to reach high volumetric capacitance for the electrodes. Finally, the high conductivity of NiCo_2_O_4_ enables fast electron transport within the electrodes to enhance the rate capability. Due to the aforementioned synergistic effects of NiCo_2_O_4_ nanoarrays and the anchored electroactive nanomaterials, the enhancement of energy storage performance of heterogeneous electrode is significant. Consequently, the advantages of highly conductive NiCo_2_O_4_ core and short ion transport channels in self‐supported 1D heterogeneous nanoarrays together with the synergistic effect between NiCo_2_O_4_ and the shell electroactive materials result in a high specific capacitance, excellent rate capability, and long cycling stability in NiCo_2_O_4_ based 1D heterogeneous nanoelectrodes. Hence, the core‐shell heterostructure and the synergistic effect undoubtedly offer an attractive synthesis strategy to further design high‐performance supercapacitor electrodes based on pseudocapacitive materials.

### 3.2 Self‐Supported Heterogeneous 2D Nanoarrays

**Figure 13:**
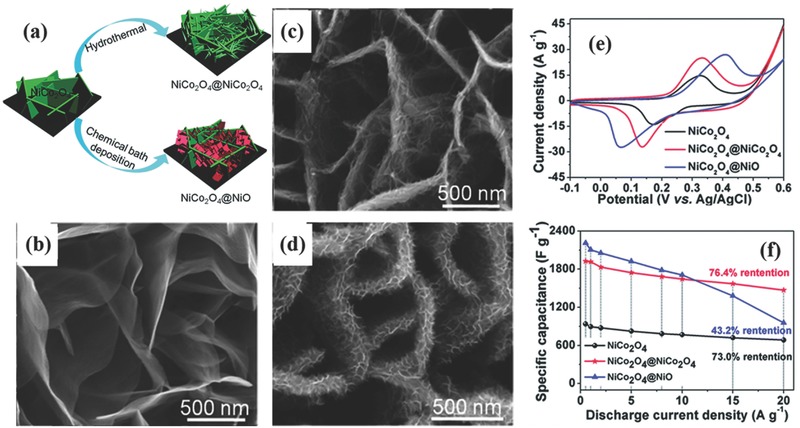
a) Schematic illustration of synthesizing homogeneous NiCo_2_O_4_ nanosheet@NiCo_2_O_4_ nanorod and heterogeneous NiCo_2_O_4_ nanosheet@NiO nanoflake core‐shell nanoarrays. SEM images of (b) bare NiCo_2_O_4_ nanosheets, (c) NiCo_2_O_4_ nanosheet@NiCo_2_O_4_ nanorod, and (d) NiCo_2_O_4_ nanosheet@NiO nanoflake core‐shell nanoarrays. e) CV curves and f) specific capacitance of the bare NiCo_2_O_4_ nanosheets, NiCo_2_O_4_@NiCo_2_O_4_ and NiCo_2_O_4_@NiO core‐shell nanoarrays. Reproduced with permission.^179^ Copyright 2014, Royal Society of Chemistry.

Similar to 1D nanoarrays as supporter and current collector for heterogeneous nanoelectrodes, template‐freely synthesized 2D nanoarrays of some metals and electroactive materials have been explored as scaffold to fabricate heterogeneous nanoelectrodes.^174^, ^175^, ^176^, ^177^, ^178^ It is clear that the open space of the 2D nanoarrays reduces the internal resistance and facilitates the diffusion of electrolyte to the inner part of the electrode when assembled to a supercapacitor. When further combined with other electroactive materials, the unique features of 2D nanoarrays make the high capacitance and excellent rate capability be highly expected for the heterogeneous nanoelectrodes attributing to the potentially synergistic effect. As aforementioned, self‐supported NiCo_2_O_4_ nanosheets have been regarded as a promising homogeneous nanoelectrode for supercapacitors, and they also represent a platform with great promise for producing heterogeneous nanoelectrodes. For example, with the pre‐formed free‐standing NiCo_2_O_4_ nanosheets on Ni foam employed as secondary nanostructured substrates, Zhou et al. fabricated intriguing homogeneous NiCo_2_O_4_@NiCo_2_O_4_ and heterogeneous NiCo_2_O_4_@NiO core‐shell nanoarrays, respectively.^179^ By adopting different solution‐phase synthesis (hydrothermal or chemical bath deposition) and following thermal decomposition methods, either NiCo_2_O_4_ nanorods or NiO nanoflakes were anchored onto the surfaces of the NiCo_2_O_4_ nanosheets (**Figure** **13**a). Figure 13b–d show the morphology of the bare NiCo_2_O_4_ nanosheets, NiCo_2_O_4_ nanosheets@NiCo_2_O_4_ nanorods, and NiCo_2_O_4_ nanosheets@NiO nanoflakes, respectively. As expected, in these hierarchical nanoarrays, the open and mesoporous nanoarchitecture of free‐standing NiCo_2_O_4_ nanosheet arrays could efficiently shorten ion transport distance to ensure a high utilization of the electrode materials and fast electrolyte irrigation, meanwhile both the core and shell materials possess good pseudocapacitive behavior to be capable of contributing to the total electrochemical charge storage. Overall, these hierarchical nanoarrays exhibited remarkable electrochemical performance as supercapacitor electrodes in terms of high specific capacitance, good rate capability, and cycling stability. Compared to NiCo_2_O_4_ nanosheet@NiCo_2_O_4_ nanorod core‐shell nanoarrays, the relative lower rate capability of NiCo_2_O_4_ nanosheet@NiO nanoflake core‐shell nanoarrays can be attributed to the lower electrical conductivity of NiO (Figure 13f). Furthermore, Qu et al. prepared more complex heterogeneous nanoelectrodes based on self‐supported Co_3_O_4_ nanosheets grown on Ni foam, Co_3_O_4_ nanobrush‐graphene@Ni_x_Co_2x_(OH)_6x_ nanoflake heterogeneous nanoelectrodes.^180^ The difference is that thin graphene film was firstly decorated on the surface of the pre‐formed Co_3_O_4_ nanobrushs before depositing Ni_x_Co_2x_(OH)_6x_ nanoflakes. The thin graphene film here is considered to efficiently increase the conductivity of Co_3_O_4_ nanobrushs and it also works as the electron transport highway for both the Co_3_O_4_ nanobrushs and Ni_x_Co_2x_(OH)_6x_ nanoflakes, benefiting for rate performance.

**Figure 14:**
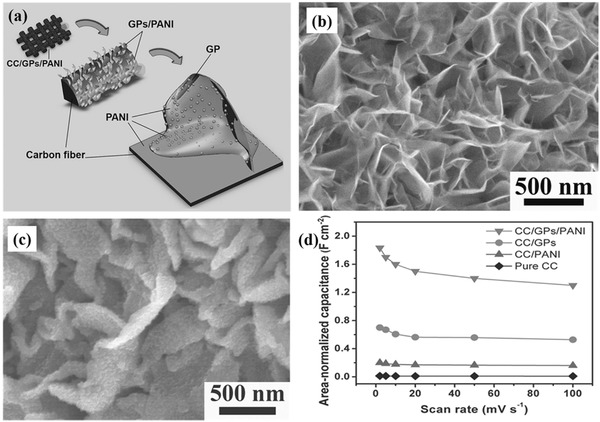
a) Schematic illustration of CC/GPs/PANI nanostructures. SEM images of (b) CC/GPs and (c) CC/GPs/PANI. d) Comparison of area‐normalized specific capacitance of pure CC, CC/GPs, CC/PANI, and CC/GPs/PANI electrodes at different scan rates. Reproduced with permission. Reproduced with permission.^181^

Another example of 2D nanoarrays as scaffold to fabricate heterogeneous nanoelectrodes is the aforementioned VG. As introduced in the homogenous 3D nanoelectrodes, the VG grown on conductive substrates is the promising building block for constructing supercapacitors with ultrahigh rate capability. In fact, the high specific surface area of VG and the high intrinsic electrical conductivity of graphene suggest the VG grown on conductive substrates to be capable of employing as nanostructured current collectors. As an example, Xiong et al. first grown graphitic petals (GPs, which is one kind of VG) on conductive carbon clothes (CC), and subsequently nanoscale thin polyaniline (PANI) was coated on surface of GPs by an electrochemical polymerization process, yielding hybrid CC/GPs/PANI electrodes (**Figure** **14**a).^181^ Apparently, the thin and smooth 2D unwrinkled GP surfaces became roughened after PANI conformally coating but still kept the original morphology of GPs (Figure 14b and 14c). Figure 14d shows the comparison of area‐normalized specific capacitances of pure CC, CC/GPs, CC/PANI and CC/GPs/PANI, respectively, at different scan rates. Obviously, the pure CC has negligible specific capacitance contribution to the electrodes, i.e. 0.01 F cm^–2^ at a scan rate of 2 mV s^–1^. After decorating GPs on CC, the specific capacitance of the composite electrode reaches 0.7 F cm^–2^ at a scan rate of 2 mV s^–1^ and decreased slightly with increasing scan rate. As CC/GPs coated with PANI, the specific capacitance of the CC/GPs/PANI sample reached 1.84 F cm^–2^ at the same scan rate, nearly one order of magnitude higher than that of CC/PANI (0.19 F cm^–2^). The hybrid electrodes also exhibited a high rate capability with an energy density of 110 Wh kg^–1^, a maximum power density of 265 kW kg^–1^ at a current density of 100 A g^–1^, and an excellent long‐term stability. With the CC/GPs/PANI as electrodes, an all‐solid state flexible supercapacitor was further fabricated with a symmetric configuration. Significantly, both the energy density (3.4 mWh cm^–3^) and the power density (3 W cm^–3^) of the device are much higher than those of the typical lithium thin‐film batteries. Likewise, Co_3_O_4_ was conformally deposited on the VG supported by carbon fabric, and then a highly flexible all‐solid‐state symmetric supercapacitor device was fabricated, delivering a high energy density of 80 Wh kg^–1^ and a high power density of 20 kW kg^–1^ (at 27 Wh kg^–1^).^182^

### 3.3 Self‐Supported 3D Nanoporous Heterogeneous Nanoeletrodes

In addition to 1D and 2D heterogeneous nanoelectrodes, there are also self‐supported 3D nanoporous heterogeneous nanoelectrodes produced by depositing electroactive materials onto 3D nanoporous current collectors that are prepared through either template‐assisted or template‐free methods.

#### 3.3.1 Template‐Free Method

**Figure 15:**
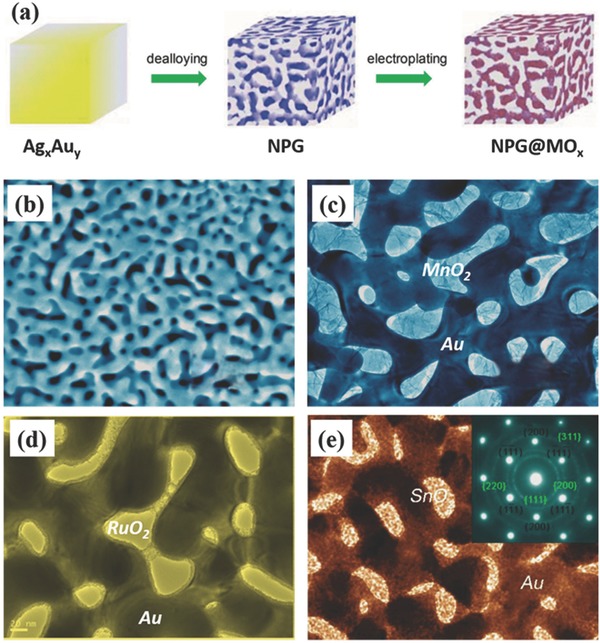
a) Schematic illustrating the fabrication procedure for NPG based heterogeneous nanoelectrodes. Reproduced with permission.^190^ Copyright 2015, Royal Society of Chemistry. SEM images of (b) as de‐alloyed nanoporous gold films, (c) MnO_2_@NPG,^184^ Reproduced with permission, Copyright 2010, Nature Publishing Group. d) RuO_2_@NPG,^185^ and e) SnO_2_@NPG.^186^ Reproduced with permission.^185^, ^186^

Different from nickel foam with the pore size in micrometer range, nanoporous metals are such a kind of metallic materials with 3D interconnected backbones and pores on the nanometer scale, possessing intriguing properties including high specific surface area and good electrical conductivity, thus to generate enormously promising potentials as 3D nanostructured current collectors for electrochemical energy storage applications.^183^ Dealloying, also known as selective dissolution, is a facile template‐free approach to generate nanoporous metals. As a typical example, Lang et al. fabricated a nanoporous gold (NPG) film via dealloying Ag_65_Au_35_ (at%) using nitric acid, followed electroplating electroactive to construct 3D heterogeneous nanoelectrodes (**Figure** **15**a).^184^ The obtained NPG film revealed a bicontinuous nanoporous structure consisting of quasi‐periodic gold ligaments and nanopore channels (Figure 15b). Although MnO_2_ has intrinsically low conductivity that restrains the realization of its high theoretical capacitance, especially at the charge/discharge rate, the NPG/MnO_2_ hybrid electrode (Figure 15c) provides good electronic/ionic conductivity to improve the reaction kinetics of MnO_2_ thin films. As a result, the NPG/MnO_2_ hybrid electrode achieved a specific capacitance as high as 1145 F g^–1^ for MnO_2_ that is close to its theoretical value (≈1370 F g^–1^), meanwhile, the energy and powder densities of the supercapacitor device were reported to be 57 Wh kg^–1^ and 16 kW kg^–1^, respectively. Similarly, NPG/RuO_2_ 185 (Figure 15d) and NPG/SnO_2_ 186 (Figure 15e) hybrid electrodes were reported with ultrahigh specific capacitance, high power density, and high energy density. For the above two cases, the excellent capacitive performance also results from the highly conductive and NPG that provides excellent electronic/ionic conductivity and large active materials/electrolyte interfaces to take full advantage of the pseudocapacitive materials. Moreover, the strong and ductile metal network could dramatically improve the mechanical rigidity and electrochemical stability of the hybrid electrodes for excellent electrochemical cycling stability. Besides metal oxides,^184^, ^185^, ^186^, ^187^, ^188^, ^189^, ^190^ metal hydroxides such as Co(OH)_2_ and Ni(OH)_2_ 191, 192 had also been decorated by NPG to enhance their pseudocapacitive behavior.^193^ With NPG/RuO_2_ as the negative electrode and NPG/Co(OH)_2_ as the positive electrode, Chen et al. introduced an asymmetric supercapacitor.^193^ Owing to advantages of NPG, both the two electrodes had comparable high specific capacitances of 1300–1800 F g^–1^, which offered the NPG/RuO_2_//NPG/Co(OH)_2_ asymmetric supercapacitor with a large capacitance of 350 F g^–1^. Consequently, the high specific capacitance and the complementary working potential window (1.6 V) gave rise to the high energy density (120 Wh kg^–1^) and high power density (70 kW kg^–1^) of the device. Other than metal oxides/hydroxides, the unique features of NPG make it easily adopt with conductive polymers, e.g., PPy,^194^, ^195^, ^196^ polyaniline (PANI).^197^ By decorating NPG with ultrathin (8 nm in thickness) PPy, Meng et al. introduced a sub‐micrometer‐thick all‐solid‐state supercapacitor with extraordinary volumetric capacitance and high power/energy densities.^194^

**Figure 16:**
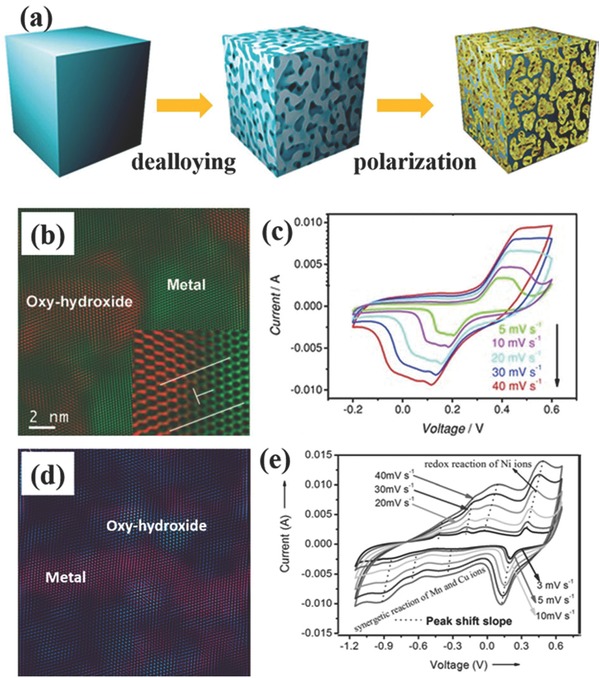
a) Illustration of the fabrication process of the oxyhydroxide supported by interconnected metal skeletons as heterogeneous nanoelectrodes.^201^ b) IFFT‐STEM atomic image displaying the distribution of the self‐grown oxy‐hydroxide and metal skeleton of the electrochemically polarized nanoporous NiMn alloy and (c) its corresponding CV curves at different scan rates. Reproduced with permission.^199^ d) The inverse FFT image showing the distribution of oxyhydroxide and metal skeletons of the electrochemically polarized nanoporous NiCuMn alloy and (e) its corresponding CV curves at different scan rates.^201^ Reproduced with permission.^199^, ^201^

It is worth noting that noble metals like gold and platinum are too expensive to limit their commercial applications in supercapacitors, thus non‐noble nanoporous metals such as nickel and copper are more economically competitive for practical supercapacitor applications.^198^ According to above consideration, the Chen group reported a facile approach to fabricate low‐cost transition‐metal based oxy‐hydroxide@nanoporous metal hybrid electrode with a nanoporous Ni‐Mn alloy (**Figure** **16**a).^199^, ^200^ The nanoporous Ni‐Mn alloy with pore size of 6–8 nm was formed by electrochemically dealloying of Ni_30_Mn_70_ alloy, and the energy dispersive X‐ray spectroscopy (EDS) analysis revealed that there were ≈14 at% Mn and ≈86 at% Ni in the obtained nanoporous Ni‐Mn alloy. After further electrochemical polarization treatment, a thin Ni‐Mn oxy‐hydroxide layer was homogeneously self‐grown in the nanopore channels of nanoporous Ni‐Mn alloy (Figure 16b). This hybrid electrode exhibited high volumetric capacitance and excellent rate capability (Figure 16c). Also based on dealloying NiMn alloy and the following electrochemical polarization treatment, Fujita et al. invented a novel mass‐producible method to prepare nanoporous metal papers for making hierarchical nanoporous Ni‐Mn electrodes.^200^ Furthermore, by dealloying and further polarization treatment of NiCuMn alloy, Kang et al. fabricated multicomponent NiCuMnOOH oxyhydroxide/nanoporous alloy (Figure 16d) as supercapacitor electrode.^201^ Originating from multiple redox reactions, the multicomponent oxyhydroxide had a high specific capacitance of 627 F cm^–3^ (1097 ± 95 F g^–1^) at a current density of 0.25 A cm^–3^, and more importantly, possessed an extraordinarily wide working potential window of 1.8 V in an aqueous electrolyte (Figure 16e).

**Figure 17:**
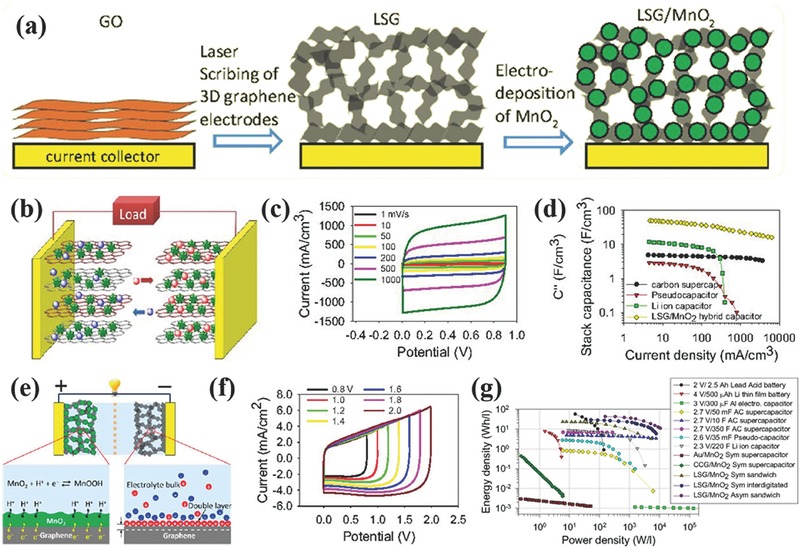
a) Schematic illustrating the fabrication procedure for the LSG‐MnO_2_ electrodes. b) Schematic diagram of the symmetric supercapacitor device based on LSG‐MnO_2_ electrodes, and its (c) CV curves as well as (d) specific capacitance. e) Schematic diagram of the asymmetric supercapacitor device based on LSG and LSG‐MnO_2_ electrodes, and its (f) CV curves as well as (g) energy/power density compared with other energy storage devices. Reproduced with permission.^90^ Copyright 2015, National Academy of Sciences.

In addition to nanoporous metals, the aforementioned highly conductive and high‐surface‐area 3D nanoporous and macroporous graphene framework is another 3D scaffold for fabricating heterogeneous nanoelectrodes.^98^, ^202^, ^203^, ^204^ El‐Kady et al. reported the fabrication of LSG‐MnO_2_ 3D nanoelectrodes by depositing MnO_2_ on the LSG framework produced from the laser scribing of graphene oxides (GO) films on a substrate (**Figure** **17**).^90^ The LSG is 3D interconnected graphene networks with high surface area and high electronic conductivity. As shown in Figure 17a, the conducting graphene framework acts as a 3D current collector to provide electron “superhighways” for charge storage and delivery, while the nanostructured MnO_2_ provides more active sites for the Faradaic reactions and shortens the ion diffusion pathways, both for realizing its full pseudocapacitance. A symmetric supercapacitor pouch cell assembling from two LSG‐MnO_2_ electrodes showed nearly rectangular CV profiles up to a scan rate as high as 1000 mV s^–1^, indicating excellent charge storage characteristics and ultrafast response time for the electrodes (Figure 17b and 17c). And, the LSG‐MnO_2_ supercapacitor showed superior volumetric capacitance of 50 F cm^–3^ and rate capability, compared with commercially available activated carbon supercapacitors, pseudocapacitors, and lithium‐ion hybrid capacitors (Figure 17d). Furthermore, an asymmetric supercapacitor was assembled using LSG‐MnO_2_ as the positive and LSG as the negative electrode, as schematically illustrated in Figure 17e. Obviously, the asymmetric configuration can extend the operating voltage window to maximum 2 V (Figure 17f) in aqueous electrolyte, leading to significantly higher specific energy than symmetric supercapacitors. Accordingly, the LSG‐MnO_2_ hybrid supercapacitors store about 6 times the capacity of state‐of‐the‐art commercially available EDLC carbon supercapacitors. And impressively, the LSG‐MnO_2_ supercapacitors can provide power densities up to ≈10 kW L^–1^, which is 100 times faster than high‐power lead acid batteries and 1000 times faster than a lithium thin‐film battery (Figure 17g).

#### 3.3.2 Template‐Assisted Method

**Figure 18:**
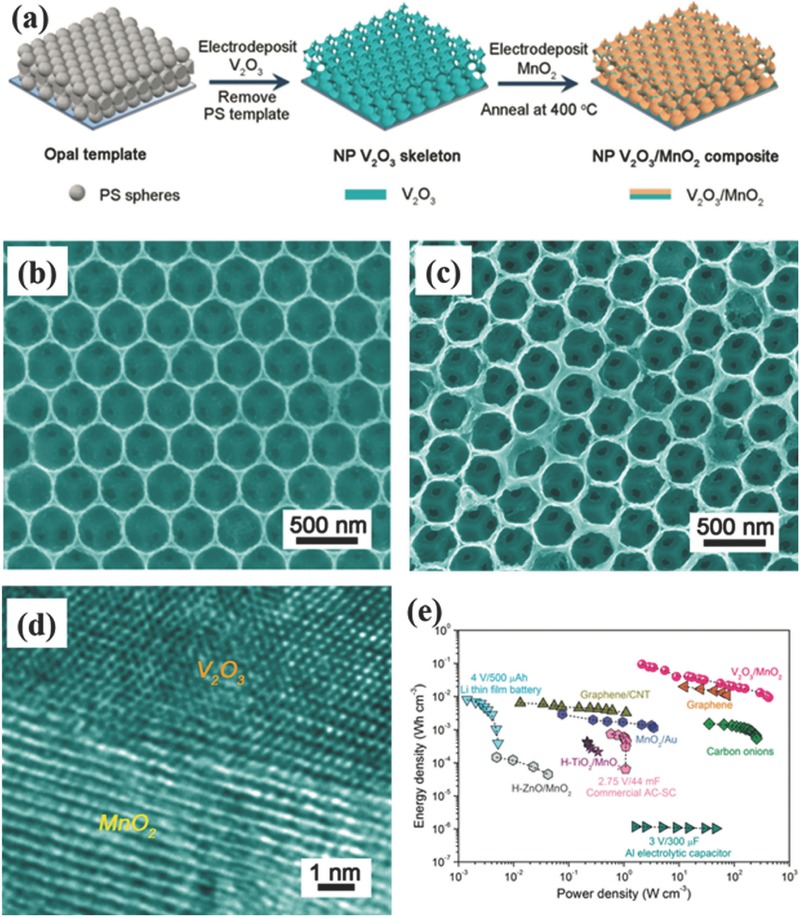
a) Scheme of preparing 3DOM V_2_O_3_/MnO_2_ electrode. SEM images of (b) 3DOM V_2_O_3_ scaffold and (c) V_2_O_3_/MnO_2_ heterogeneous nanoelectrode. d) HRTEM image of V_2_O_3_/MnO_2_ interfacial structure. e) The comparison of volumetric energy and power densities of V_2_O_3_/MnO_2_‐based pesudocapacitors with other energy storage devices. Reproduced with permission.^210^

Template‐synthesis is another popular way to prepare 3D nanoporous current collectors.^205^ Hereinto, the three‐dimensionally ordered macroporous (3DOM) metals prepared by a colloidal crystal templating method is one of promising 3D nanoporous architecture as current collector for supercapacitors. Apart from the feature of large and conductive surface, the ordered interconnected pore network of 3DOM enables rapid ion transport in the electrolyte‐filled pores greater than in the circuitous pore structure in dealloying‐derived chaotically nanoporous metals, and then would be more favorable for high rate capability of supercapacitors. For example, 3DOM of Mn/MnO_2_,^206^ Ni/Cu_2_O,^207^ and Ni/NiO^208^ core/shell electrode, respectively, with a shell inverse‐opal structure via electrodeposition method with monodisperse polystyrene spheres as template. Owing to the high conductivity of core Mn layer that offers an electron avenue for charge transfer reaction to overcome the key drawback (low electric conductivity) of MnO_2_ and the high surface area to enable rapid, reversible Faradaic reactions, a very impressive specific capacitance over 1200 F g^–1^ for MnO_2_ had been finally obtained. With potential sweep rate increasing from 5 to 500 mV s^–1^, the specific capacitance of 3DOM Mn/MnO_2_ core/shell electrode was 996 F g^–1^, which is 83% of the capacitance maintained relative to that measured at 5 mV s^–1^, indicating the better rate capability of 3DOM Mn/MnO_2_ core/shell electrode. Very recently, Li et al. fabricated 3DOM Ni/MnO_2_ onto the surface of a Ni wire as flexible fiber electrodes.^209^ Benefiting from the structural merits of the designed electrode configuration, the obtained solid‐state fiber supercapacitor had achieved high energy and power density as well as long cycle life and high rate capability. In addition, Lang et al. found that 3DOM structure of vanadium sesquioxide (V_2_O_3_) could also serve as current collector because V_2_O_3_ with corundum‐type crystalline structure becomes highly conductive at ambient temperature as a consequence of metallization via insulator‐to‐metal transition.^210^ The fabrication of 3DOM V_2_O_3_ also made use of polystyrene spheres as template and MnO_2_ was selected as the example of pseudocapacitive materials (**Figure** **18**a). Figure 18b and 18c are the representative top‐view SEM images of 3DOM V_2_O_3_ before and after electroplating MnO_2_, respectively. It is evident that MnO_2_ nanocrystals uniformly grown along the walls of 3DOM V_2_O_3_ while the whole structure still keep a 3D bicontinuous nanoporous structure. High‐resolution transmission electron microscope image (Figure 18d) indicates the epitaxial growth of MnO_2_ nanocrystals on V_2_O_3_ core skeleton surfaces and the formation of V–O–Mn bonding at the interface. The abundant V_2_O_3_/MnO_2_ epitaxial interfaces could dramatically enhance both the interfacial electron transfer via V–O–Mn chemical bonding and the electrical conductivity of the poor conductive MnO_2_ layer by the formation of semiconductive region. Consequently, these unique features in combination with the advantages of 3DOM structure allowed a symmetric supercapacitor based on 3DOM V_2_O_3_/MnO_2_ electrodes to deliver exceptionally high volumetric power density (≈422 W cm^–3^) and volumetric energy density (≈94 mWh cm^–3^), which are five orders and one order of magnitude higher than that of lithium thin‐film battery (4 V/500 µAh), respectively (Figure 18e), meanwhile with excellent cycling stability over 15000 cycles. Also with polystyrene spheres as sacrificial template, Choi et al. prepared 3D macroporous graphene framework as supporter/current collector and subsequently functionalized with MnO_2_ to use as a supercapacitor electrode.^99^ Attributing to the porous structure, large surface area and high conductivity of 3D porous graphene framework, the heterogeneous nanoelectrodes was endowed with excellent electrochemical properties such as a specific capacitance of 389 F g^–1^ at 1 A g^–1^ and a rate capability with 97.7% capacitance retention upon a current increase to 35 A g^–1^. Additionally, the asymmetrical supercapacitor assembled based on 3D porous graphene with and without MnO_2_ coating as the two electrodes had shown remarkable supercapacitor performance: wide cell voltage of 2 V, energy density of 44 Wh kg^–1^, power density of 25 kW kg^–1^, and excellent cycle life.

**Figure 19:**
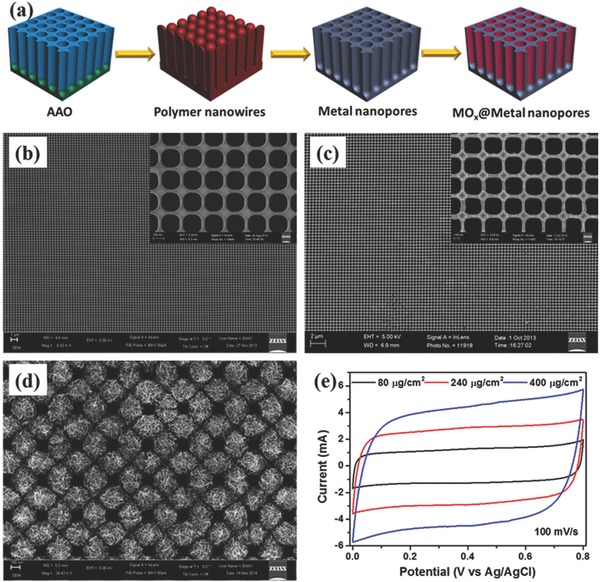
a) Schematic illustration of the fabrication process of metallic nanopores based heterogeneous nanoelectrodes. SEM images of (b) original AAO template and (c) as‐fabricated Ni nanopores. d) SEM image of Ni nanopores after being coated with MnO_2_ (400 µg cm^–2^). e) CV curves of MnO_2_@Ni nanopores with different MnO_2_ loadings at a scan rate of 100 mV s^–1^. Reproduced with permission.^211^

With respect to design an ideal supercapacitor electrode with larger specific surface area and meanwhile lower ion transport resistance, the structure features of AAO template (e.g., highly‐oriented nanoporous structure and large surface area) is believed to be of great referential significance. Considering the insulator nature of alumina, metallic nanopore arrays with exactly the same structure of the AAO structure was fabricated using a two‐step replication process to utilize as 3D nanostructured current collectors for constructing heterogeneous nanoelectrodes (**Figure** **19**a).^211^ The whole fabrication process involves (1) polymer infiltration into AAO template and the following removal of AAO template to obtain polymer nanorod arrays; (2) electroplating Ni with polymer nanorods as template and the further dissolution of polymer to obtain metallic nanopores; (3) electrodeposition of electroactive materials to produce heterogeneous nanoelectrodes. The obtained AAO‐like Ni nanopore arrays have exactly the same structure as the original AAO template (Figure 19b and 19c). With such metallic nanopore arrays as a nanostructured current collector to construct core/shell type supercapacitor electrodes, the higher specific surface area and the lower ion transport resistance can be achieved simultaneously. The large specific surface area could ensure high capacitance, while the highly oriented nanoporous structure can facilitate ion transport; thus high‐performance supercapacitors have been realized. Moreover, the structure of nanopore arrays can be considered as the negative structure of nanowire arrays. Ideally, when both nanopore and nanowire arrays are synthesized from the same AAO template, they will have the equivalent specific surface area if they have the same diameter and length. But unlike nanowire arrays that will agglomerate with increasing aspect‐ratio, the metallic nanopore arrays are actually a nanoporous metal film which is a self‐supported and homogeneous structure, thus would have much better mechanical stability. The increasing aspect‐ratio of nanopore arrays will only result in the increasing specific area, which will benefit for improving the mass loading meanwhile still keep lower ion transport resistance, and consequently will enhance the energy density as well as the rate capability of supercapacitors. If nanopores with larger diameters are used as current collector, it will offer not only larger specific surface area for the coating of active materials, which is helpful for increasing the mass loading of active materials per unit substrate area. At the same time, the highly oriented nanoporous structure could be maintained to facilitate the ion transport after being coated with even more active materials, e.g., MnO_2_ mass loading raising from 80 to 400 µg cm^–2^ (Figure 19d), attributing to the larger nanopore diameter, and then will increase the energy storage capability of electrode (Figure 19e). Accordingly, with metallic nanopore arrays as current collectors for constructing heterogeneous nanoelectrodes, high capacitance, excellent rate capability, and long cycling stability could be principally achieved simultaneously.

## 4 Conclusions and Outlook

In this Review, we have summarized the recent progress in design and fabrication of self‐supported supercapacitor nanoelectrodes with various architectures and compositions through either template‐free or template‐assisted synthesis methods, and highlight their advantages for constructing supercapacitors with high energy storage capability in terms of high energy density, high power density, excellent rate capability, and long cycling stability. The aim of design and fabrication of self‐supported supercapacitor nanoelectrodes is just to meet the critical requirements, such as enhanced ion/electron conductivity, faster reaction kinetics and stronger mechanical stability, to enhanced energy storage capability of supercapacitors. As discussed above, most of electroactive materials have therefore been fabricated into a variety of self‐supported nanoarrays with different geometry (including 1D nanostructures such as nanowire, nanotube, and nanorod; 2D nanostructures such as nanosheet, nanowall, and nanoflake; as well as 3D macro‐/nanoporous structure) onto various conductive substrates as binder‐ and additive‐free electrodes for supercapacitors. Generally, all the reported self‐supported nanoelectrodes for supercapacitors can be classified into homogeneous and heterogeneous nanoelectrodes as outlined in Scheme 1. Typical examples of reported homogeneous and heterogeneous nanoelectrodes (including electroactive materials, morphology, synthesis method, specific capacitance, cycling performance, and electrolytes) have been summarized in **Table** **1**. In comparison with the conventional slurry‐casting supercapacitor electrodes, both homogeneous and heterogeneous nanoelectrodes possess superiorities as binder‐free electrodes to achieve high specific capacitance, excellent rate capability, and long cycling stability, greatly attributing to their larger ion‐accessible specific surface area, lower electronic/ionic transport resistance, and robust adhesion on conductive substrates. With respect to the approaches for fabricating self‐supported nanoelectrodes, it can be basically categorized into template‐free and template‐assisted approaches, involving the fabrication techniques like electrochemical deposition, chemical vapor deposition, atomic layer deposition, hydrothermal method, electrospinning, annealing, and chemical bath deposition, etc. Template‐free approaches are usually simple and feasible but less controllable in the structural uniformity and porosity of the obtained nanoarrays. On the contrary, the self‐supported nanoelectrodes from template‐assisted approaches have controllable and predefined structure/porosity depending on the employed sacrificial templates, however, a removable template with desirable nanometric scale features is certainly required leading to a rather complicated fabrication process, especially more for heterogeneous nanoelectrodes. Notably in Table 1, although the fabrication of heterogeneous nanoelectrodes involves tedious synthesis procedures and is normally high production cost when compared to the fabrication of homogeneous nanoelectrodes, heterogeneous nanoelectrodes enable to boost the electrochemical performance of pseudocapacitive materials especially transition metal oxides (who has higher theoretical pseudocapacitance but lower electrical conductivity) because of the complementary and/or synergistic effects in addition to all the advantages of homogeneous nanoelectrodes, allowing the pseudocapacitive materials to approach their theoretical potentials in supercapacitors (e.g. high energy density at high power density). Thereby, the construction and optimization of heterogeneous nanoelectrodes should become one of the research focuses in the field of supercapacitors.

**Table 1:** Typical examples of reported homogeneous and heterogeneous nanoelectrodes

Self‐supported nanoarraysa)	Synthesis methodb)	Electrolytec)	Capacitance	Ref.
1D Homogeneous nanoelectrodes				
mesoporous MnO_2_ nanowires	AAO template + ED	0.5 m Na_2_SO_4_	493 F g^–1^ at 4 A g^–1^; 97.3% capacitance retention after 800 cycles	22
PANI nanowires	AAO template + ED	2 m H_2_SO_4_	1142 F g^–1^ at 5 A g^–1^; 95% capacitance retention after 500 cycles	26
MnO_2_ nanotubes	AAO template + ED	1 m Na_2_SO_4_	320 F g^–1^ at 20 mV s^–1^; 81% capacitance retention after 2000 cycles	30
RuO_2_ nanotubes	AAO template + ED	1 m H_2_SO_4_	1300 F g^–1^ at 100 mV s^–1^	32
PEDOT nanotubes	AAO template + ED	1 m LiClO_4_	140 F g^–1^ at 100 mV^–1^	33
TiN nanotubes	AAO template + ALD	1 m KOH	167 F g^–1^ at 1 A g^–1^; 85% capacitance retention after 6000 cycles	34
carbon nanotubes	AAO template + CVD	6 m KOH	72 F g^–1^ at 2 A g^–1^	36
3D RACNTs	AAO template + CVD	PVA‐H_2_SO_4_	89.4 mF cm^–2^ or 23.9 mF cm^–1^; no capacitance change over 10000 cycles	37
Ni(OH)_2_ nanotubes	ZnO template + hydrolysis	1 m KOH	1328 F g^–1^ at 1 A g^–1^	39
PANI nanotubes	ZnO template + ED	1 m H_2_SO_4_	675 F g^–1^ at 50 mV s^–1^; 70% capacitance retention after 400 cycles	40
carbon nanotubes	ZnO template + CVD	6 m KOH	182 F g^–1^ at 40 A g^–1^; nearly 100% capacitance retention after 20000 cycles	41
TiN nanocorns	Co_2_(OH)_2_CO_3_ template + ALD + annealing	1 m LiClO_4_	20.7 F cm^–3^ at 1 V s^–1^; nearly 100% capacitance retention after 20000 cycles	44
NiCo_2_O_4_ nanoneedles	Solvent method + annealing	2 m KOH	3.12 F cm^−2^ at 1.11 mA cm^–2^; 89.3% capacitance retention after 2000 cycles	53
ZnCo_2_O_4_ nanorods	Solvent method + annealing	1 m KOH	1400 F g^–1^ at 1 A g^–1^; 97% capacitance retention after 1000 cycles	56
Bamboo‐like carbon nanofibers	Electrospinning + annealing	3 m KOH	236 F g^–1^ at 5 A g^–1^; nearly 100% capacitance retention after 5000 cycles	63
V_2_O_5_ nanofibers	Electrospinning + annealing	2 m KCl	190 F g^–1^ at 5 mV s^–1^	64
1D Heterogeneous nanoelectrodes				
RuO_2_@MnO_2_ nanowires	AAO template + ED + annealing	1 m Na_2_SO_4_	302 F g^–1^ at 20 mV s^–1^; 90% capacitance retention after 5000 cycles	28
Co_3_O_4_@MnO_2_@NiO nanotubes	AAO template + annealing	1 m KOH	1224.5 F g^–1^ at 12.2 A g^–1^; 80% capacitance retention after 5700 cycles	35
MnO_2_@ZnO nanowires	ZnO template + ED	1 m Na_2_SO_4_	405 F g^–1^ at 10 mV s^–1^	38
(Cu,Ni)O mesoporous nanowires	Hydrothermal + annealing	3 m NaOH	4.28 F cm^–2^ at 1 mA cm^–2^; 100.9% capacitance retention after 6000 cycles	59
MnO_2_@Ni nanorods	AAO template + ED	0.5 m K_2_SO_4_	190 F g^–1^ at 10 mV s^–1^	111
NiO@Ni nanowires	AAO template + ED + annealing	1 m KOH	179 F g^–1^ at 10 mV s^–1^; 80% capacitance retention after 2000 cycles	112
MnO_2_@Pt nanotubes	AAO template + ALD + ED	1 m Na_2_SO_4_	810 F g^–1^ at 5 mV s^–1^; nearly 100% capacitance retention after 8000 cycles	114
MnO_2_@TiN nanotubes	AAO template + ALD + ED	1 m LiClO_4_	662 F g^–1^ at 45 A g^–1^	115
MnO_2_@SnO_2_nanotubes	AAO template + ALD + ED	1 m Na_2_SO_4_	910 F g^–1^ at 1 A g^–1^	116
MnO_x_@Au nanocones	AAO + ED	1 m Na_2_SO_4_	840.3 F g^–1^ at 2 A g^–1^; 96.5% capacitance retention after 2000 cycles	119
MnO_2_@ZnO nanowires	ZnO template + ED	0.5 m Na_2_SO_4_	138.7 mF cm^–2^ at 1 mA cm^–2^; 87.5% capacitance retention after 10000 cycles	122
ZnO nanorod@NiO/MoO_2_ nanosheets	ZnO template + hydrothermal	2 m KOH	1.18 F cm^–2^ at 5 mA cm^–2^; 91.7% capacitance retention after 4000 cycles	123
MnO_2_–NiO nanoflakes	ZnO template + hydrothermal + annealing	1.5 m LiOH	0.35 F cm^–2^ at 50 mV s^–1^; 96.4% capacitance retention after 1600 cycles	124
PPy@Ni nanotubes	ZnO template + ED	5 m LiCl	474.4 F g^–1^ at 5 mV s^–1^; 75.3% capacitance retention after 10000 cycles	127
α‐Fe_2_O_3_@PPy nanowires	ZnO template + vapor phase polymerization	1 m Na_2_SO_4_	382.7 mF cm^–2^ at 0.5 mA cm^–2^; 97.2% capacitance retention after 5000 cycles	132
Coaxial TiN‐VN nanofibers	Electrospinning + annealing	1 m KOH	185 F g^–1^ at 20 mV s^–1^; 88% capacitance retention after 500 cycles	134
CoO nanowires@MnO_2_ nanosheets	Hydrothermal + annealing	KOH‐PVA	1835 F g^–1^ at 1 A g^–1^; 97.7% capacitance retention after 10000 cycles	153
Co_3_O_4_ nanowire@MnO_2_ nanosheets	Hydrothermal	1 m LiOH	480 F g^–1^ at 2.67 A g^–1^; 97.3% capacitance retention after 5000 cycles	158
Co_3_O_4_@Co(OH)_2_ nanosheets	ED + annealing	2 m KOH	1095 F g^–1^ at 1 A g^–1^; 92% capacitance retention after 2000 cycles	160
2D Homogeneous nanoelectrodes				
Co_3_O_4_ nanosheets	ED + calcination	2 m KOH	2735 F g^–1^ at 2 A g^–1^; 99% capacitance retention after 3000 cycles	71
VO_2_ nanoflakes	Solvothermal	1 m K_2_SO_4_	485 F g^–1^ at 2 A g^–1^	73
Ni_3_S_2_ nanosheets	Hydrothermal sulfidization	1 m NaOH	1000 F g^–1^ at 50 A g^–1^; nearly 100% capacitance retention after 20000 cycles	74
MnO_2_ nanosheets	Hydrothermal	1 m KOH	595.2 F g^–1^ at 0.5 A g^–1^; 89% capacitance retention after 3000 cycles	76
NiCo_2_O_4_ nanosheets	CBD + annealing	2 m KOH	3.51 F cm^–2^ at 1.8 mA cm^–2^; 6.7% capacitance loss after 3000 cycles	77
VG	PECVD	6 m KOH	156 F g^–1^ at 100 A g^–1^	97
NiCo_2_O_4_ nanograsses	ED + calcination	3 m KOH	2.13 F cm^–2^ at 1 mA cm^–2^; 80% capacitance retention after 3000 cycles	163
NiCo_2_S_4_ nanopetals	Hydrothermal	1 m KOH	2036.5 F g^–1^ at 1 A g^–1^; 94.3% capacitance retention after 5000 cycles	170
^2D Heterogeneous nanoelectrodes^				
Cu@Ni(OH)_2_ nanobelts	CBD+galvanic displacement	1 m KOH	2426 F g^–1^ at 10 A g^–1^; 91% capacitance retention after 1000 cycles	174
NiCo_2_O_4_@Co_x_Ni_1−x_(OH)_2_ nanosheets	ED + annealing	1 m KOH	5.71 F cm^–2^ at 5.5 mA cm^–2^; 83.3% capacitance retention after 3000 cycles	175
CuCo_2_O_4_ nanosheets@MnO_2_ nanoflakes	Hydrothermal + annealing	1 m Na_2_SO_4_	416 F g^–1^ at 1 A g^–1^; 92.1% capacitance retention after 4200 cycles	159
Co_2_AlO_4_@MnO_2_ nanosheets	Hydrothermal	2 m KOH	99.13 F g^–1^ at 2 A g^–1^; 96.1% capacitance retention after 2000 cycles	178
NiCo_2_O_4_ nanosheets @NiCo_2_O_4_ nanosheets	Hydrothermal	3 m KOH	1925 F g^–1^ at 0.5 A g^–1^; 90.1% capacitance retention after 3500 cycles	179
NiCo_2_O_4_ nanosheet@NiO nanoflakes	CBD	3 m KOH	2210 F g^–1^ at 0.5 A g^–1^; 85.4% capacitance retention after 2000 cycles	179
Co_3_O_4_@Ni_x_Co_2x_(OH)_6x_ nanobrushs	Hydrothermal + ED	1 m LiOH	2550 F g^–1^ at 1 A g^–1^; 99.15% capacitance retention after 5000 cycles	180
Co_3_O_4_ nanoparticles@VG	Hydrothermal	2 m KOH	3482 F g^–1^ at 1 mV s^–1^; 86.2% capacitance retention after 20000 cycles	182
3D Homogeneous nanoelectrodes				
LSG	Laser irradiation	1 m H_2_SO_4_	4.04 mF cm^–2^ at 1 A g^–1^; 96.5% capacitance retention after 10000 cycles	89
3D graphene hydrogels	Hydrothermal reduction	PVA‐H_2_SO_4_	372 mF cm^–2^ at 1 A g^–1^; 91.6% capacitance retention after 10000 cycles	92
3D holey graphene	Hydrothermal	6 m KOH or EMIMBF_4_‐AN	298 F g^–1^ at 1 A g^–1^; 91% capacitance retention after 10000 cycles	94
Holey graphene oxide hydrogels	Reduction induced solution self‐assembly	1 m H_2_SO_4_	283 F g^–1^ at 1 A g^–1^; 90% capacitance retention after 20000 cycles	95
3D macroporous graphene	PS template + solution method	1 m Na_2_SO_4_	93 F g^–1^ at 1 A g^–1^	99
TiC hollow spheres	PS template + ALD + annealing	1 m EMIMBF_4_	291 F g^–1^ at 12 A g^–1^; 98% capacitance retention after 75000 cycles	100
3D Heterogeneous nanoelectrodes				
MnO_2_@LSG	Laser scribing + ED	1 m Na_2_SO_4_	1136.5 F cm^–3^ at 1 mV s^–1^; 96% capacitance retention after 10000 cycles	90
MnO_2_@3D graphene	Ni foam template + CVD + ED	0.5 m Na_2_SO_4_	1.42 F cm^–2^ at 2 mV s^–1^	98
MnO_2_@3D macroporous graphene	PS template + solution method + ED	1 m Na_2_SO_4_	202 F g^–1^ at 1 A g^–1^; 95% capacitance retention after 1000 cycles	99
MnO_2_@NPG	Dealloying + Electroless plating	2 m Li_2_SO_4_	1145 F g^–1^ at 50 mV s^–1^; 85% capacitance retention after 1000 cycles	184
RuO_2_@NPG	Dealloying + ED	0.5 m H_2_SO_4_	1450 F g^–1^ at 20 A g^–1^	185
SnO_2_@NPG	Dealloying + Electroless plating	2 m Li_2_SO_4_	75 F cm^–3^ at 1.25 A cm^–3^; 92% capacitance retention after 30000 cycles	186
MnO_2_@NPG	Dealloying + ED	EMI‐DCA	160 F g^–1^ at 8 A g^–1^; 81% capacitance retention after 1000 cycles	187
MnO_2_@NPG	Dealloying + ED	1 m Na_2_SO_4_	922 F cm^–3^ at 5 mV s^–1^; 88% capacitance retention after 20000 cycles	188
PPy@NPG	Dealloying + ED	HClO_4_‐PVA	270 F g^–1^ at 0.6 A g^–1^; 78% capacitance retention after 900 cycles	194
Oxy‐hydroxide@NPM	Dealloying	1 m KOH	505 F cm^–3^ at 0.5 A cm^–3^	199
NiCuMnOOH@NPM	Dealloying + Polarization	1 m KOH	627 F cm^–3^ at 0.25 A cm^–3^; 99.7% capacitance retention after 2300 cycles	201
CoMoO_4_@3D graphene	Hydrothermal + annealing	2 m KOH	2741 F g^–1^ at 1.43 A g^–1;^ 96.36% capacitance retention after 10000 cycles	202
3DOM Mn/Mn oxide	PS template + anodiziation	3 m KCl	1260 F g^–1^ at 2 A g^–1;^ 89% capacitance retention after 2000 cycles	206
3DOM Cu_2_O/Ni	PS template + ED + annealing	6 m KOH	502 F g^–1^ at 10 mV s^–1^	207
3DOM Ni–NiO	PS template + ED + annealing	1 m KOH	10 mF cm^–2^ at 0.2 mA cm^–2^	208
3DOM V_2_O_3_@MnO_2_	PS template + ED	1 m Na_2_SO_4_	1162 F cm^–3^ at 1.56 A cm^–3^; 86% capacitance retention after 15000 cycles	210
MnO_2_@Ni nanopore arrays	AAO template + ED	1 m Na_2_SO_4_	672 F g^–1^ at 2 mV s^–1;^ 83% capacitance retention after 3000 cycles	211

^a)^ Nanoporous gold: NPG; Nanoporous metal: NPM

^b)^ Electrochemical deposition: ED; chemical vapor deposition: CVD; chemical bath deposition: CBD; atomic layer deposition: ALD. plasma‐enhanced chemical vapor deposition: PECVD

^c)^ poly(vinyl alcohol): PVA; acetonitrile: AN; 1‐ethyl‐3‐methylimidazolium tetrafluoroborate: EMIMBF_4_; 1‐ethyl‐3‐methylimidazolium dicyanamide: EMI‐DCA.

While self‐supported nanoelectrodes exhibit many advantages and the performance of supercapacitors based on self‐supported nanoelectrodes has been significantly improved, there are still several important issues that need to be addressed in order to fully exploit their potentials for real supercapacitor applications. First, compared to slurry‐casting electrodes, the practical applications of self‐supported nanoelectrodes are still greatly hampered by the low active‐mass loading per unit electrode area in nanoelectrodes, which is one of the most challenging issues of nanoelectrodes not only for supercapacitors but also for batteries applications. In order to fabricate practical devices, the electroactive material mass loading per electrode area needs to be more than 1 mg cm^–2^, however, the mass loading of most nanoelectrodes are only in the range of several tens to several hundreds of microgram. Although high specific capacitance, even very close to the theoretical value, has been successfully achieved in nanoelectrodes with low mass loading, nonetheless, the low active‐mass loading per unit electrode area leads to a corresponding low electrode capacitance, resulting in low energy density of the electrode and the following supercapacitor device. Finally, the low device energy density makes the nanoelectrodes‐based supercapacitor only ideal as micropower sources for some low power microsystems. Aiming at improving the device energy density to further broaden the applications, one of the research concern about self‐supported nanoelectrodes should be focusing on developing efficient strategies to significantly increase the mass loading in self‐supported nanoelectrodes, and meanwhile, the principal merits of supercapacitors based on self‐supported nanoelectrodes such as high power density, high rate capability, and long cycling stability must not be sacrificed. Second, from the device prospective, the merits of self‐supported nanoelectrodes would bring fully beneficial effects if they could be retained when they are assembled into a full supercapacitor device. To reach this goal, the device configuration and the choice of counter electrodes, electrolytes, and separators, as well as the device packaging technique have to be intensively investigated and optimized, and meanwhile the systematic research about the mechanical integrity and electrochemical kinetics of electrodes/devices is also fundamentally needed. Regarding the high energy density, asymmetric supercapacitors with self‐supported nanoelectrodes as both the positive and the negative electrodes is more advantageous because of the wider potential window, leading to higher energy density according to *E* = *0.5CV^2^*. It is noted that to achieve the maximum asymmetric supercapacitor device capacitance, the charge storage capacity of both positive and negative electrodes has to be properly balanced. Recently, the new‐concept battery‐supercapacitor hybrid devices have been attracted great interest.^212^, ^213^, ^214^, ^215^, ^216^, ^217^ Different from the conventional asymmetric supercapacitors that both electrodes are capacitive, a battery‐supercapacitor hybrid device consists of one battery electrode and one capacitive electrode (either EDLC or pseudocapacitive), thus is expected to possess the best features of both supercapacitors (high power density and excellent rate capability) and batteries (high energy density). It is generally believed that sluggish redox processes occur in battery‐type electroactive materials and thus hinder the power performance. Besides discovering new high‐performance materials for battery‐supercapacitor hybrid devices, the design and construction of self‐supported nanoelectrodes as both battery‐electrode and capacitive electrode would be able to boost the electrochemical kinetics of both battery electrode and capacitive electrode, and finally allow to reach battery‐like energy density and supercapacitor‐like power density of the hybrid device. With respect to electrolytes, gel or polymer electrolytes are especially preferred. When gel or polymer electrolytes fully infiltrating into the open interspaces of self‐supported nanoelectrodes, it will enable intimate interfacial contact between electroactive material and electrolyte, and at the same time the gel or polymer electrolyte could act as curing agent to “freeze” the arrayed structure features of self‐supported nanoelectrodes, thus avoiding the structural degradation of nanoarrays during the cycling to largely enhance the long‐term cycling stability. Last, it is well known that scale‐up production of self‐supported nanoelectrodes with high throughput way is crucial for commercial applications. Although many aforementioned fabrication strategies are potentially or principally scalable, it still should be aware that the synthetic routes might be hard to be economically implemented in large scale because of some unexpected technological challenges, and thus the scale‐up fabrication of self‐supported nanoelectrodes with low cost and high throughput should be also of research concern.

Overall, with the continuous achievements in advanced electroactive materials, optimized electrode design, and novel device assembling technologies, self‐supported nanoelectrodes based new‐generation supercapacitors with superior energy storage capability can be highly expected for wider technological applications.

## Conflict of Interest

The authors declare no conflict of interest.
